# Modeling Evaluations of Low-Level Sounds in Everyday Situations Using Linear Machine Learning for Variable Selection

**DOI:** 10.3389/fpsyg.2020.570761

**Published:** 2020-10-23

**Authors:** Siegbert Versümer, Jochen Steffens, Patrick Blättermann, Jörg Becker-Schweitzer

**Affiliations:** Institute of Sound and Vibration Engineering, University of Applied Sciences Düsseldorf, Düsseldorf, Germany

**Keywords:** machine learning, variable selection, human perception, situation, Lasso, environmental sound, online-survey

## Abstract

Human sound evaluations not only depend on the characteristics of the sound but are also driven by factors related to the listener and the situation. Our research aimed to investigate crucial factors influencing the perception of low-level sounds as they—in addition to the often-researched loud-level sounds—might be decisive to people’s quality of life and health. We conducted an online study in which 1,301 participants reported on up to three everyday situations in which they perceived low-level sounds, resulting in a total of 2,800 listening situations. Participants rated the sounds’ perceived loudness, timbre, and tonality. Additionally, they described the listening situations employing situational eight dimensions and reported their affective states. All sounds were then assigned to the categories natural, human, and technical. Linear models suggest a significant difference of annoyance ratings across sound categories for binary loudness levels. The ability to mentally fade-out sound was the most crucial situational variable after valence, arousal, and the situation dimensions positivity and negativity. We ultimately selected the most important factors from a large number of independent variables by applying the percentile least absolute shrinkage and selection operator (Lasso) regularization method. The resulting linear regression showed that this novel machine-learning variable-selection technique is applicable in hypothesis testing of noise effects and soundscape research. The typical problems of overfitting and multicollinearity that occur when many situational and personal variables are involved were overcome. This study provides an extensive database of evaluated everyday sounds and listening situations, offering an enormous test power. Our machine learning approach, whose application leads to comprehensive models for the prediction of sound perception, is available for future study designs aiming to model sound perception and evaluation.

## Introduction

Myriad research has shown that annoyance reactions to unpleasant sounds can cause psychological stress (Gunn et al., 1975; Wolsink et al., 1993; Lercher, 1996; Stallen, 1999) that consequently affects cognition and health (Serrou, 1995; Babisch, 2002; World Health Organization, 2011; Beutel et al., 2016; Klatte et al., 2017). While the majority of studies have focused on the perception, evaluation, and effects of medium or loud sounds generated by road traffic (Aletta et al., 2018; Riedel et al., 2019) and aircraft noise (Kroesen et al., 2008; Schreckenberg et al., 2016), annoyance has also been found in response to low-level sounds—for example, noise from wind turbines (Wolsink et al., 1993; Crichton et al., 2015; Klatte et al., 2017; van Renterghem, 2019). Since research to date in the field of wind turbine noise has focused on low frequencies, we aimed to investigate the evaluation of low-level day-to-day sounds in general and to establish comprehensive models including situational, sound-related, and person-related factors to predict the perception of environmental sounds in both low- and mid/high-level scenarios. Moreover, we investigated which influencing factors had a substantial impact on the evaluation of low-level sounds when taking into account multiple variables. To address these research aims, we conducted an online study wherein 1,301 participants reported on up to three everyday situations (including 32 relevant sound-related, situational, and person-related variables) in which they perceived low-level sounds. To handle this large number of variables, we implemented the percentile least absolute shrinkage and selection operator (Lasso) method, a linear machine learning approach, to select the crucial variables associated with annoyance ratings and to establish comprehensive models which overcome problems associated with overfitting and can predict annoyance for new data that were not involved in the model training and validation.

Previous research on soundscape perception and reactions to noise has identified several influencing factors related to sound, situation, and perception. Besides exposure level (Wolsink et al., 1993; Basner et al., 2014; Guski et al., 2017), these factors include many non-auditory variables, such as sensitivity to noise (Fields, 1993; Job, 1996; Schreckenberg et al., 2010; Hill et al., 2014; Shepherd et al., 2015; Park et al., 2016; Kim et al., 2017), extraversion and neuroticism with contradictory evidence for the relevance of this factor (Lercher, 1996), attitude toward the source or the authorities that operate the sound source (Stallen, 1999; Job et al., 2007; Kroesen et al., 2008), perceived disturbance (Stallen, 1999; Kroesen et al., 2008), fear of the noise source (Miedema and Vos, 1999), and the failure to cope with the environment which leads to stress (Guski, 1999). Many objective situational variables, including the presence of other people, the location of the perceiver, the sound insulation of dwellings, the visibility of the source, economic benefit through the source, exposure time, or ambient noise level (Fields, 1993; Wolsink et al., 1993; Bangjun et al., 2003; Janssen et al., 2011; Steffens et al., 2017) have also been identified as relevant factors.

### Psychological Situations and Situational Characteristics

Situations can be seen as “fluctuating, dynamic, and dependent upon different perspectives” (Rauthmann, 2015, p. 177). Since situational factors are known to be essential predictors of human perception and behavior, they have been the subject of many studies. Nevertheless, these factors, interpreted as “situational,” have mostly been physical, objective, easily measurable, and (in a laboratory setting) controllable quantities: exposure time, noise insulation of dwellings, and ambient noise (Fields, 1993); age, benefit, and visibility of the source (Bangjun et al., 2003; Janssen et al., 2011); or exposure level, buildings, trees, and fences (Wolsink et al., 1993). Situations may be defined by the actual objective environment (*E*) and the momentary mental and affective state of the person (*P*) perceiving the specific situation (*S*). Lewin (1936) described a person’s behavioral states (*B*) driven by a function of the perception of that situation as *B* = f(*P*, *E*) = f(*S*). Following this theory, situations can be split up into cues, characteristics, and classes (Rauthmann et al., 2015). The objective physical quantities mentioned above can be seen as the situational cues from which people derive situational characteristics and psychological meaning during the evaluation processes. Finally, situational classes group situations that have similar characteristics or cues.

This view of situations is in line with the model of the “cognitive–motivational–emotive system” discussed by Smith and Lazarus (1990, p. 622). In that model, objective conditions—the cues—are individually interpreted by the person through imprinting his or her personality, including individual needs, commitments, goals, knowledge, attitudes, and beliefs. The resulting subjective situational construal—the characteristics—ultimately serves as the basis for the subsequent appraisal processes that mediate a person’s emotional response. For example, imagine a bike path parallel to a highly frequented 8-lane road surrounded by tall trees in full leaf. Cyclists who were highly skeptical of the greenery’s capability to attenuate traffic noise, improve air quality, or enhance health reported lower soundscape quality (Aletta et al., 2018).

The importance of taking psychological and situational characteristics into account is evident, as they reflect situational social aspects and people’s cognitive and emotional perceptions of their environments. To propose a taxonomy for measuring and describing psychological situations, Rauthmann et al. (2015) developed the DIAMONDS model through measuring individual differences in situation perception. This model consists of eight situational dimensions: “*Duty* (does something need to be done?), *Intellect* (is deep information processing required?), *Adversity* (is someone being overtly threatened?), *Mating* (is the situation sexually and/or romantically charged?), *pOsitivity* (is the situation pleasant?), *Negativity* (do negative things taint the situation?), *Deception* (is someone deceptive?) and *Sociality* (is social interaction and relationship formation possible, desired, or necessary?)” (Rauthmann et al., 2015, p. 364). The DIAMONDS model follows the principle of personality research that “individuals [may] think about situational characteristics in much the same way they think about personal characteristics” (Halevy et al., 2019, p. 4). Interestingly, to the best of our knowledge, such a model has not yet been used to investigate sound evaluation in terms of differences in individual situation perception. Therefore, we included the assessment of psychological situations in our study and hypothesized that psychological situation characteristics would significantly be associated with annoyance ratings of environmental sounds.

### Stress and Its Precursors as Pivotal Points in Human Perception

In addition to the psychological situations that might play an essential role in human perception of sound, perceived control was assumed to be “the most important non-acoustical determinant of environmental noise annoyance” in the stress–annoyance model developed by Stallen (1999, p. 77), which interpreted annoyance as stress. He hypothesized that annoyance is driven by three main factors: perceived disturbance, perceived control, and coping with annoyance.

The first factor, perceived disturbance, depends on the sound of the sources and an initial cognitive–emotive appraisal process (Lazarus, 1966; Stallen, 1999). Disturbance occurs when people are hindered from achieving their goals (e.g., concentration, relaxation, sleep, communication). It is linked to annoyance both directly (Stallen, 1999; Kroesen et al., 2008) and indirectly through a mediated path via coping strategies (Park et al., 2016). Disturbance is also influenced by the personality trait noise sensitivity (Park et al., 2016).

The second factor, perceived control, is not associated with disturbance or noise (Stallen, 1999). Instead, it has been hypothesized that perceived control is driven by the noise management of the source—not the source itself—and that it directly affects annoyance through a secondary path (Lazarus, 1966; Stallen, 1999; Park et al., 2016). Kroesen et al. (2008) followed the approach of Stallen’s stress model in investigating annoyance induced by aircraft noise (Stallen, 1999). Perceived control and coping capacity were together shown to be the most important variables after concerns about adverse health effects and perceived disturbance.

Coping can be defined as “constantly changing cognitive and behavioral efforts to manage specific external and/or internal demands that are appraised as taxing or exceeding the resources of the person” (Lazarus and Folkman, 1984, p. 141). Coping is driven by “the belief and confidence of an affected person that he/she will somehow manage the problem” (Guski, 1999, p. 51). Coping with stress in general or annoyance in particular can be seen as a reappraisal of a person’s environment (Gunn et al., 1975; Smith and Lazarus, 1990; Stallen, 1999). Botteldooren and Lercher (2004), as well as Glass et al. (1972), assumed that annoyance is a prerequisite for coping. Park et al. (2016) reported an additional mediation effect of coping on the relationship between disturbance and annoyance that was not present in the model by Kroesen et al. (2008).

### Personality Traits and Demographic Factors

In contrast to the aforementioned dynamic situational factors, stable personality traits change little in adulthood. The Big Five dimensions of personality, for example, were derived through a lexical approach, meaning that all relevant aspects of personality will develop and be found in the language of a community: *Neuroticism*, *Extraversion*, *Openness to experience*, *Agreeableness*, and *Conscientiousness* have been consistently reported to be important and sufficient descriptors of human personality (for an overview, see Digman, 1990; Costa and McCrae, 2008). Though *Extraversion* and *Neuroticism* (as well as all demographic variables) have often been discussed in relation to human sound evaluation, the results have been controversial (see the review by Fields, 1993). Even when all Big Five dimensions are considered together, a recent study by Lindborg and Friberg (2016) showed only small, albeit significant, effects.

Noise sensitivity seems to play an essential role in moderating or mediating the effect of sound on annoyance (Miedema and Vos, 2003; van Kamp et al., 2004) and health (Job, 1996). Shepherd et al. (2015) analyzed the effect of (other) personality traits on sensitivity to noise and revealed that extraversion acted as a major predictor. In their study, all Big Five dimensions showed linear and independent effects on noise sensitivity and together accounted for 33% of variance. Similarly, Lindborg and Friberg (2016) reported that noise sensitivity can be predicted by extraversion and conscientiousness. Belojević and Jakovjlevic (2001) also investigated factors influencing sensitivity to noise and found that neuroticism was the only significant person-related factor in noisy environments but had no significant effect in quiet areas. Since noise sensitivity plays a vital role in sound perception and since extraversion and neuroticism may influence noise sensitivity, extending existing findings by investigating these variables for low-level sounds seems worthwhile.

Demographic variables have often been investigated in noise annoyance research, showing only a small or generally insignificant effect (Yu and Kang, 2008). Miedema and Vos (1999) reported that people between 20 and 70 years of age showed higher annoyance compared to younger or older people. Gender was not significant, but education level showed a small effect of increased annoyance with increasing years of education. The hypothesis that people with higher education, and thus higher income, experience less annoyance by seeking less noisy living environments seems to apply only to residents of small or medium-sized cities, with income not significantly moderating annoyance (Fyhri and Klćboe, 2006).

### Aims and Hypotheses

Many of the studies mentioned above have focused on a small number of variables associated with sound evaluations and annoyance reactions. Our study, in contrast, combined a high number of relevant sound-related, situational, and person-related variables in a comprehensive model to predict low- and mid/high-level sounds in everyday life. We therefore attempted to identify the most relevant predictors. Based on previous research, we assumed that situational variables, as opposed to person-related factors, would have higher explanatory potential in predicting annoyance ratings of both low- and mid/high-level sounds. We further hypothesized that the category of a sound (natural vs. technical vs. human) would play a decisive role in evaluating environmental sounds. We included demographic factors to investigate the extent to which previous results are reproducible in a retrospective online study. Finally, we explored which low-level sounds participants perceived as particularly pleasant or annoying and how often these sounds occurred in day-to-day life.

## Materials and Methods

### Participants

Initially, we defined 18 quotas of 100 participants each. The quotas were established by combining three *Age Class*es (20–40, 41–60, and 61–80 years) with two *Gender*s (*female* and *male*) and three *Education Level*s (International Standard Classification of Education (ISCED) levels 0–2 (up to lower secondary education), level 3 (upper secondary education), and levels 4–8 (university-level education); United Nations Educational, Scientific and Cultural Organization (UNESCO) UNESCO Institute for Statistics, 2015. For the application to the German education system (see Schneider, 2008). We commissioned the Cologne-based commercial market research company respondi AG^1^ to provide suitable participants from its online panel according to our quota targets defined above.

Of the 12,000 persons invited by email, 4,087 started the online questionnaire; 1,815 (54%) completed the questionnaire and reported and evaluated 5,445 sound situations. Of these, 514 (28%) reported no sound situations or gave implausible answers. Consequently, 2,645 datasets were excluded from the evaluation. We ultimately analyzed the data of 1,301 participants (630 men, mean age = 49.9, *SD* = 15.5; 671 women, mean age = 49.6, *SD* = 16.4), who reported on 2,800 sound situations. *Education Level* and *Gender* were evenly distributed (men: 223 level 3, 185 below, 222 above; women: 228 level 3, 196 below, 247 above). In addition to German subjects, the sample also included a few respondents of non-German *Nationality* (12 men; 13 women). Participants with any type of *Hearing Impairment* (*n* = 213; 16.4%) were included in the final dataset since the mean *Annoyance* ratings of all their reports did not differ significantly from the ratings by persons without a known hearing disability [Wilcoxon (*W*) = 530,871; *p* = 0.749; calculated on raw data]. Additionally, their *Noise Sensitivity* (*M* = 15.16; *SD* = 3.76; calculated on raw data) did not differ significantly (*W* = 107,813; *p* = 0.107) from participants without a known *Hearing Impairment* (*M* = 14.70; *SD* = 4.13).

### Procedure

To address our research topics regarding the occurrence of low-level environmental sounds in everyday life and person-related and situational factors influencing their evaluations, we conducted a large-scale online study using the LimeSurvey^2^ software (see the questionnaire in the Supplementary Material). After reporting on sociodemographic and person-related variables, participants described and evaluated up to three sound situations they had experienced in the past, with no acoustic stimuli provided by us. (If a participant reported less than three sound situations, we provided one to three preset sound situations, so that each participant had three to evaluate. The situations we added were not taken into account in this analysis.) We let the participants decide how they understood “quiet” or “low-level” (in the sense of “not loud”). We further used the term “sound” to avoid bias toward negatively perceived sounds classified as “noise.” After finishing the questionnaire, the participants were automatically redirected to the panel operator respondi AG to receive monetary compensation for their participation.

### Design and Questionnaire

In our online study, we asked the participants to remember and evaluate low-level sounds they had heard. Thus, we focused on sound immission and not on sound emission. Since perceived sound level decreases with increased distance from the source and can be changed in terms of frequency components, we believe that evaluating sound sources from a greater distance—e.g., through closed windows—will lead to biased, experience-driven judgments. The questionnaire we used in our study is provided as Supplementary Material.

#### Person-Related Variables

Besides the sociodemographic variables (i.e., *Age*, *Gender*, *Education Level, and Nationality*), participants reported other temporal stable variables, such as whether they were aware of having any *Hearing Impairment*. They further rated their living environment by answering the question, “How would you describe your living environment?” using the five-level bipolar item *Liveliness*, ranging from *very lively* (1) to *very calm* (5). They also reported on the number of *Persons* living in the household (*1* to “*6 or more*”) and their household’s monthly disposable *Net Income* (German Federal Statistical Office, 2018). Moreover, since the person-related factors *Extraversion*, *Neuroticism*, and *Noise Sensitivity* have shown associations with sound evaluations in previous studies (Fields, 1993; Job, 1996; Lercher, 1996; Schreckenberg et al., 2010; Hill et al., 2014; Shepherd et al., 2015; Kim et al., 2017), we obtained those factors using the German 10 Item Big Five Inventory (BFI-10; Rammstedt and John, 2007; Rammstedt et al., 2012) and a nine-item *Noise Sensitivity* questionnaire (“Kurzform des Fragebogens zur Lärmempfindlichkeit,” LEF-K, developed by Zimmer and Ellermeier, 1998). Participants also answered the question “Are you generally able to mentally fade out sounds (even loud ones)?” using a five-level scale ranging from *does not apply at all* (0) to *is absolutely right* (4) for the item *General Fade-out*. To group the reports for each participant (see section “Statistical Analyses” for the random effect), we assigned a unique *ID* to each participant.

#### Sound-Related Variables

In addition to the temporally stable person-related variables, the following variables are assumed to change over time depending on the sound and its embedding situation. Participants responded to the item “Please remember sounds that you have classified as low-level in your environment in the past.” by reporting sounds in free-form text descriptions. They also rated the perceived *Loudness* of their sounds (“How do you rate the sound?”) on a five-level scale ranging from *scarcely audible* (1) to *low-level* (3) to *middle and louder* (5). The *Loudness* levels 4 and 5 were intended to check whether participants had indicated a low-level sound. The *Timbre* of the sound was assessed on a five-level bipolar scale ranging from *deep, dull* (1; German: “tief, dumpf”) to *high, shrill* (5; German: “hoch, schrill”) as well as the item *Tonality* based on the levels *broadband noise* (1; German: “rauschartig”) to *tonal* (5; German: “tonhaltig”).

They also used a German translation of the standard soundscape dimensions by responding to the question “Please indicate how much you consider the following characteristics to be a description of the sound.” These eight dimensions—namely *Pleasant* (“angenehm”), *Vibrant/Exciting* (“lebendig/pulsierend”), *Eventful* (“ereignisreich”), *Chaotic* (“chaotisch”), *Annoying/Distracting* (“lästig/störend”), *Monotonous* (“monoton”), *Uneventful* (“ereignislos”), and *Calm* (“ruhig”)—are measured on Likert scales ranging from *strongly agree* (1) to *strongly disagree* (5) and can be arranged in a circumplex model of soundscape perception (Axelsson et al., 2010; Lindborg and Friberg, 2016; following ISO/TS 12913-2, ISO, 2018). To obtain the dependent variable *Annoyance* that was relevant for our analyses, we computed the arithmetic mean across the ratings of *Pleasant* (inversed) and *Annoying*.

#### Situational Variables

Concerning the time-varying situational variables, participants responded to “For each sound, please mention a situation in which you have experienced this sound.” using free-form text descriptions. Participants’ affective state (“Please assess how you feel in this sound situation.”) was obtained in terms of *Valence* (*negative-positive*), *Arousal* (*calm-excited*), and the perceived *Control* over the sound situation (*weak-strong*). Here, we used the Self-Assessment Manikin (SAM), which consists of three sets of nine pictograms (see the questionnaire in Supplementary Material) representing the different states of the three affective dimensions (Lang, 1980; Bradley and Lang, 1994; PXLab: Irtel, 2007). The SAM has been shown to be quickly and consistently answerable by people of various nationalities and languages, by adults and children, and by people with language disorders (Bradley and Lang, 1994; Bynion and Feldner, 2017). It has also been demonstrated to be applicable to the evaluation of acoustic stimuli (IADS: Bradley and Lang, 2000) and therefore seemed suitable for our study. The use of the SAM can activate responses in any part of the emotional system, like physiological, behavioral, and emotional (Suk, 2006). Thus, the SAM seems to be a more profound measurement method than written scales, which must be processed via cognition. The pictograms can be modified or replaced with signs to achieve similar results (Affective Slider: Betella and Verschure, 2016). In many studies, the original SAM was adapted in terms of number of levels, number of pictograms, and manipulation of the pictograms (Bynion and Feldner, 2017; Bartosova et al., 2019). The semiotics of the pictograms and signs, although self-explanatory, are usually explained at the beginning of a test (Lang and Bradley, 1997; Suk, 2006). Võ et al. (2009) used the SAM to avoid the German translation for arousal (“Erregung”), which could have sexual associations. Since the SAM pictograms can be used for many attributes other than valence, arousal, and dominance (Suk, 2006), we believed that additional descriptions of the three affective variables were necessary. Because slightly modified words were successfully used in most studies, we added the adjectives given above to clarify the two anchors of each of these scales.

Participants further responded to the question “Can you mentally fade out this sound?” for the *Specific Fade-out* variable using a five-level Likert scale ranging from *does not apply at all* (0) to *does fully apply* (4). To assess participants’ *Active Coping* response to the sound situation, we asked “Suppose you feel disturbed by the sound 1 in situation a^3^. Would you take action to reduce the disturbing effect?” to which respondents answered *yes* or *no*. For a more detailed description of the situation and its psychological characteristics, we utilized an ultra-brief German measure of the situational eight-factor DIAMONDS model (S8-II; Rauthmann, 2018). Finally, participants reported the *Frequency* of occurrence of the described situation using six levels: *less than once a year* (0); *once to four times per year* (1); *five to 11 times per year* (2); *once to three times monthly* (3); *once to three times weekly* (4); *four to seven times weekly* (5); and *more than once a day* (6).

### Data Analysis

#### Data Preparation

We first analyzed all sound descriptions and classified them into the three *macro-level sound categories* of *natural*, *human*, and *technical* sounds that have already been applied in previous studies (Axelsson et al., 2010; Bones et al., 2018) as well as the soundscape standard (ISO 12913-2, ISO, 2018).

We further established 38 *micro-level sound categories* (see Figure 3) in the course of a more detailed qualitative analysis. This categorization was mainly carried out using two processing loops. In the outer loop, an audio expert looked through the sound descriptions and searched for an often-mentioned sound or word. A category was then established for the sounds described by that word. This definition was based on the knowledge of sound properties, sound sources, and theories of sound perception. For example, the two categories *Dogs and insects with possible threats* and *Dogs, insects, and other animals without possible threats* were created, as an individual’s attitude to the sound source can change the perception of sound; e.g., a fly might be less annoying than a mosquito because one expects a possible painful mosquito bite. Another example was the *Signals* category, which includes all types of signals—such as ring tones, alarm clocks, and doorbells—that have a concrete meaning for the participant and urge the participant to take action. Some sounds have been combined, such as sounds caused by garbage collection and construction site noises, since participants usually have no direct influence on these sound sources. As a result, reduced perceived control and limited coping may emphasize annoyance.

In the inner loop, the data was then filtered by this word or iteratively for a part of the word (e.g., by omitting the word ending). The word was also modified or replaced by a synonym. If the context derived from the sound and situation descriptions of each filtered sound situation matched the noise category, all these reports were assigned to the selected category and excluded from further handling. By this exclusion, the number of remaining reports was reduced successively. If no further observation could be assigned to this category, the process was resumed with the outer loop and the next category was defined. These loops were repeated until all sound situations were categorized. Accordingly, there were numerous categories and no “undefined” group. Datasets which included responses with nonsensical terms (e.g., “fff”) or in a language other than German were excluded from the evaluation.

We also derived categories for the *Location* where participants experienced the described situations (see Table 1). In addition, we applied the *Activity* categories introduced by Greb et al. (2018), which we adapted slightly for our data, as shown in Table 1. Namely, we removed *Making music*, added *Making a call*, and assigned new activities to the existing categories when a similar evaluation distribution existed. Of the 13 *Activity* categories, the category *Undefined* was the largest due to 978 situation descriptions that contained no information about activities. We therefore excluded the *Activities* from further detailed analysis.

**TABLE 1:** Categories used for the variables *Location* and *Activity*.

**Description**	**Variable**	**N_obs_**
**Location**		
At home, indoors	*Home (indoors)*	1,659
At home outdoors, incl. garden, and nature	*Garden/Nature*	463
Undefined	*Undefined*	259
Other, indoors	*Other (indoors)*	212
At work/office	*Work/Office*	129
Other (outdoors)	*Other (outdoors)*	78
**Activity**		
Undefined		978
Relaxing, falling asleep, awakening		864
Being on the move, transportation		331
Working, studying, cognitive work		220
Entertainment (TV, radio, movie, theater, gaming, internet surfing)		116
Housework		88
Social activities		74
Taking a meal		50
Personal hygiene		30
Exercise, sport, leisure activities, hobbies		17
Making a call		17
Pure music listening and entertainment (TV, books/news reading)		8
Coping with emotions and stress		7

**N_obs_ Number of observations in the mentioned category.**

To test the inter-rater reliability, a second rater assigned the sound situation descriptions to the *micro-level sound categories.* For 190 descriptions, none of these categories seemed reasonable. Again, reports with nonsensical sound and situation descriptions were marked for removal. The point estimate of Krippendorff’s alpha of 0.782 with bootstrapped 95% confidence intervals (CIs) [0.767, 0.796]^4^ were obtained by bootstrapping 1,000 samples (Zapf et al., 2016). This reliability lies between α = 0.667 and α = 0.800, which is why the *micro-level sound categories* should only be used for “drawing tentative conclusions” (Krippendorff, 2004, p. 241). According to Krippendorff, an α above 0.800 is considered reliable, which we observed for the *macro-level* sound categories (α = 0.877, CI [0.863, 0.891]^5^). Values for the *Locations* (α = 0.625, CI [0.599, 0.650]^6^) are below that threshold, which allows only tentative conclusions.

As we were particularly interested in the perception and evaluation of *low-level* sounds, we grouped all datasets according to their *Loudness* rating as *low-level* (*Loudness* levels [1–3]) or *mid/high-level* ([4–5]). The person-related variable describing the perceived *Liveliness* of the living environment was grouped into the category *calm* or *lively* through a median split (*Mdn*_environment_ = 4). Finally, we assigned to each participant the mean value of the reported *Net Income* interval and combined two variables—number of *Persons* living in the household and monthly disposable household *Net Income*—to form a new variable: net *Income per Person*.

#### Statistical Analyses

All statistical analyses were performed with R 3.6.3 (R Core Team, 2020) and R-Studio (RStudio Team, 2019). To predict *Annoyance* assessments by person-related, situational, and sound-related variables, we calculated several hierarchical linear mixed-effect models, as such models can handle non-normally distributed data and take into account dependencies of the three observations (level 1) within the participants (level 2) while allowing for the inclusion of time-varying (i.e., situation-related) predictors. The participants, represented by their grouping *ID*, were included as a random factor in all models we used in this paper (see Table 2 for an overview of all models).

**TABLE 2:** Overview about the Models used in this contribution.

**Model**	**Fixed factors**	**Data**	**Presented information**	**Used in**
*Macro1*	*Macro-level sound categories*	All data	Estimated marginal means and CI	Sections “Statistical Analyses” and “Sound Categories: Macro-Level”
*Macro2*	*Macro-level sound categories* + *Binary loudness* levels			Section “Statistical Analyses” Figure 2

*Micro1*	*Micro-level sound categories*	All data	Estimated marginal means and CI	Sections “Statistical Analyses” and “Influence of Micro-Level Sound Categories”
*Micro2*	*Micro-level sound categories* + *Binary loudness*			Section “Statistical Analyses” Figure 3
*Micro3a*	*Micro-level sound categories*	*Low-level* subset		Figure A1 in the Annex
*Micro3b*	*Micro-level sound categories*	*Mid/high-level* subset		

*Location*	*Location* + binary *Loudness*	All data	Estimated marginal means and CI	Sections “Statistical Analyses” and “Influence of Location” Figure 4

*Liveliness*	*Liveliness* + binary *Loudness*	All data	Estimated marginal means and CI	Sections “Statistical Analyses” and “Living Environment” Table 3

*32SFF*	32 single-fixed-factor models	All data	β, CI, *p*, df, *R*^2^_m_, *R*^2^_c_	Sections “Statistical Analyses” and “Single-Fixed-Factor Models” Table 4
*32SFFA*	32 single-fixed-factor models (ANOVA)		β, *R*^2^_m_	Section “Statistical Analyses” and “Single-Fixed-Factor Models” Figure 5

Age*Edu	*Age* * *Education*	All data	β, CI, *p*, df, *R*^2^_m_, *R*^2^_c_	Sections “Statistical Analyses” and “Role of Person-Related Factors” Table 5

*CML1 CML2 CML3*	LASSO selected variables	All data *Low-level* subset *Mid/high-level* subset	β, CI, *p*, df for all LASSO selected variables and loudness subsets	Sections “Percentile Lasso Regression Parameter Selection Method” and “Comprehensive Models” Table 6

*CMSFF1 CMSFF2 CMSFF3*	Relevant variables from single-fixed-factor models	All data *Low-level* subset *Mid/high-level* subset	β, CI, *p*, df, R^2^_m_, R^2^_*c*_	Sections “Statistical Analyses” and “Comprehensive Models” Table 7

**SFF singe-fixed-factor; CM comprehensive model.**

To calculate these models, we used two different approaches. First, we used the *lme4* (Bates et al., 2015) and *performance*^7^ R packages to calculate the marginal and conditional coefficients of determination (*R*^2^_m_ and *R*^2^_c_) as effect size measures (models *32SFF/32SFFA*, *Age^∗^Edu*, and *CMSFF1/2/3*). *R*^2^_m_ addresses the variance of *Annoyance* that is explained by fixed factors, whereas *R*^2^_c_ represents the variance that is explained by both fixed and random factors (Nakagawa and Schielzeth, 2013). We derived probability values for each implemented variable and factor-level dummy using the *lmerTest* R package (Kuznetsova et al., 2017). To assess the influence of each variable of interest (see subsections of section “Design and Questionnaire”) on *Annoyance*, we built one single-fixed-factor model for each variable and dummy (Table 4, model *32SFF*). We also derived one probability value for each variable (or, in the case of a factor, including the dummies of a factor) using ANOVA (Figure 5, model *32SFFA*). Several publications have shown that ANOVA may be successfully applied to non-normally distributed data (Glass et al., 1972; Harwell et al., 1992; Lix et al., 1996). The probability values were calculated using Satterthwaite’s approximation of degrees of freedom. This approximation combined with restricted maximum likelihood estimation produces “the most consistent Type 1 error rates, being neither anti-conservative nor overly sensitive to sample size” (Luke, 2017, p. 1500).

Second, we used bootstrapping—drawing 50,000 samples—with the *clusterBootstrap* R package (Deen and de Rooij, 2020) to calculate the marginal means of *Annoyance*, including the 95% CIs for non-normally distributed data (models *Macro1/2, Micro1/2/3a/b*, *Location*, and *Liveliness*). This method uses linear models, is relatively free of assumptions, and is particularly well suited for hierarchical data. Non-normally distributed data were considered by resampling the observations at the individual level (within persons). This means that if one observation of a person was selected randomly, all other observations of this person were also included in the calculation (which is also the case for the Lasso regression method described in the next section).

We used β as a standardized regression coefficient only for independent variables that did not represent a physical quantity (such as age, income, and persons in the household). The factor levels represented by their dummy variables were also standardized. The dependent variable *Annoyance* was not standardized for a more intuitive interpretation.

When calculating CIs or probabilities, we accepted the inflation of Type I errors because applying a correction to the confidence levels and *p*-values for all models used in this paper would have resulted in many different confidence levels, complicating the interpretation. Additionally, reducing the family-related error rate using a correction method would have increased the probability of Type II errors and reduced the validity of the test. More importantly, the discussion of which correction should be used and how the family might be defined would be beyond the scope of this paper, as this is a very controversial topic in the research community (Rothman, 1990; Perneger, 1998; Bender and Lange, 2001). The same applies to the general use of probabilities in the context of linear mixed-effects regression models (Luke, 2017). Thus, all the probabilities and CIs given here should be interpreted in this context and should not be seen as a hard cut-off condition.

##### Percentile lasso regression parameter selection method

**FIGURE 1:**
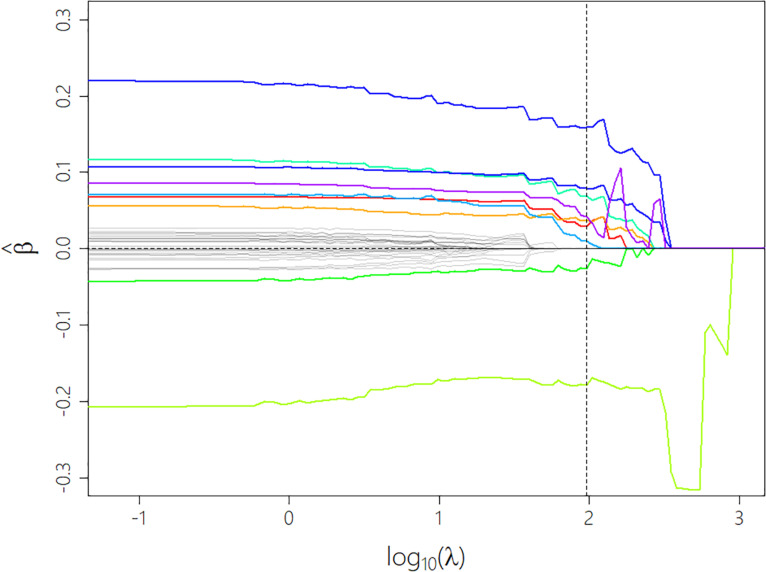
Progression of the coefficients as a function of the tuning parameter λ during the shrinking process. The colored lines show predictors that don’t get eliminated until the optimal λ (vertical dotted line) is reached. Dummy variables that constitute one factor variable share the same color. Most coefficients follow the expected decreasing trend while some (see light green curve) show a completely unexpected and sometimes even strongly transient progression which can be considered as regularization artifacts.

One of our aims was to establish a comprehensive model predicting the perceived *Annoyance* of *low-level* sounds by utilizing the most essential sound-related, situational, and person-related factors. To this end, we followed the work of Greb et al. (2018) and used the percentile-Lasso regression method (Roberts and Nowak, 2014) for multilevel linear regression modeling based on the measured data (models CML1/2/3). The Lasso method was first described by Tibshirani (1996) and has become a popular shrinkage method in the field of statistical learning algorithms. It adds a ℓ^1^ regularization term with a tuning parameter λ to linear regression models that controls the amount of shrinkage applied to the regression coefficients. Choosing a high λ value potentially sets all coefficients to zero, while λ = 0 results in a linear regression model without penalty (see Figure 1). This form of regularization can thus be used to extract important features from the data and reduce overfitting by excluding less important predictors from the model and therefore lowering its complexity. To achieve these advantages, the optimal compromise between retaining all contributing factors in the model (λ = 0) and excluding all variables (at λ_max_) must be found. Therefore, the loss function, minimized within the Lasso, is defined in Eq. 1.

(1) $$\sum_{i=1}^{n}{\left(y_{i}-{\hat{y}}_{i}\right)}^{2}=\sum_{i=1}^{n}{\left(y_{i}-\sum_{j=0}^{p}\beta_{j}\times x_{i\,j}\right)}^{2}+\lambda \,\sum_{j=0}^{p}\left|\beta_{j}\right|$$

We chose five-fold cross-validation to find the optimal λ value that results in a parsimonious and generalized model with small prediction error. The technique of *K*-fold cross-validation involves randomly splitting the data into *K* nearly equally sized folds and using *K*–1 folds as training data. The remaining fold is used for validating the previously estimated statistical model and calculating the mean squared error (MSE) of the prediction on unseen data that was not involved in the training. This routine is repeated *K* times until every fold has been used as a validation set, resulting in a cross-validation error (*CV*) as the mean MSE calculated from all *K* repetitions. Our decision that *K* = 5 resulted from the number of observations—considering computational costs and a sensible amount of data in the validation sets—since research has noted that 5- and 10-fold cross-validation can be viewed as equally efficient with regards to the bias–variance tradeoff (Krstajic et al., 2014). The random fold assignment was—respecting the two-level structure of the measured data—based on the level of the participants to assure that all measurements of one participant were assigned to either the training or validation set for all repeatitions within the cross-validation.

A set of 100 λ values (grid) was used to build and validate models within every cross-validation cycle. As suggested by Greb et al. (2018), the grid had an exponential form to achieve a higher resolution of values toward zero. The value λ_max_ was determined in advance by successively increasing λ until all regression coefficients were set to zero. As proposed by Hastie et al. (2009), the 1-SE^8^ rule was then applied to calculate the optimal λ value for every cross-validation cycle and to choose the most parsimonious model whose MSE was within one SE of the minimum cross-validation error.

To overcome the sensitivity of finding the optimal λ value to the cross-validation fold assignment (Krstajic et al., 2014), we repeated the process of cross-validation 100 times and selected the 95th percentile as the optimal λ value for the final fit. As reported by Roberts and Nowak (2014), the 95th percentile produces good and reliable results.

We used the *glmmLasso* R package (Groll, 2017) to implement the percentile-Lasso regression method. The package allowed us to calculate the generalized linear mixed effect models using a group Lasso estimator, as proposed by Groll and Tutz (2014), which applies the same amount of shrinkage to all dummy variables that constitute one factor variable. All factor variables in the dataset were coded as dummy variables. All predictor variables, including the dummy variables, were z-standardized to ensure a fair penalization and to compare their relative contributions to the *Annoyance* ratings. The factor levels containing the most observations were selected as the reference category for the dummy creation, as depicted in the caption of Table 6.

## Results

### Descriptive Statistics

#### Sound Categories: Macro-Level

**FIGURE 2:**
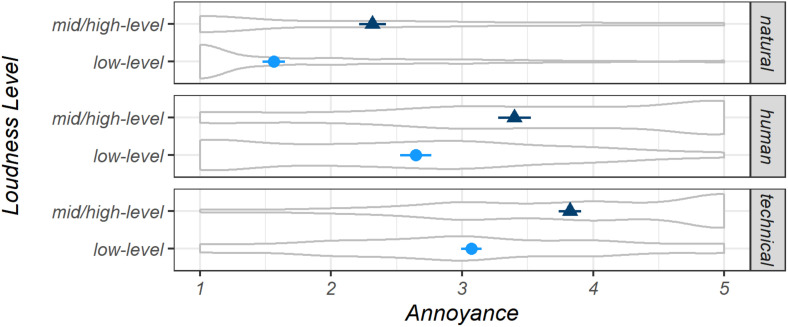
Estimated marginal means for *Annoyance* for *natural*, *human*, and *technical* sounds, separated by binary *Loudness* levels, displayed with 95% confidence intervals, both determined by bootstrapping. *Very pleasant* = 1, *very annoying* = 5. Distributions of the underlying measured *Annoyance* judgments are presented in gray. Model *Macro2*.

Participants reported 904 *natural* (32%), 552 *human* (20%), and 1,344 *technical* sounds (48%) as well as 1,260 (45%) *mid/high-level* and 1,540 (55%) *low-level* sounds (separated by the binary perceived *Loudness* variable). The results of a linear mixed-effects model (*Macro1*) revealed that predicted mean *Annoyance* of the three *macro-level sound categories* differed significantly according to the bootstrapped CIs (model *Macro1*): *M*_natural_ = 1.87, CI [1.79, 1.95]; *M*_human_ = 3.06, CI [2.94, 3.19]; *M*_technical_ = 3.41, CI [3.33, 3.48]. Figure 2 shows the estimated marginal means and CIs for both levels of perceived *Loudness*, indicating significant differences between all means (model *Macro2*). The differences of the estimated marginal mean values in relation to the *Loudness* levels were the same for all three sound categories due to the addition of *Loudness* levels as a second fixed factor. Figure 2 also displays the underlying distributions of the measured data for the subsets shown. The distributions for the *macro-level sound categories human* and *technical* differed across *Loudness* levels. As expected, more *mid/high*-level sounds were reported at higher levels of *Annoyance*. The *low-level human* sounds were in the opposite direction, and *low-level technical* sounds were normally distributed.

#### Influence of Micro-Level Sound Categories

**FIGURE 3:**
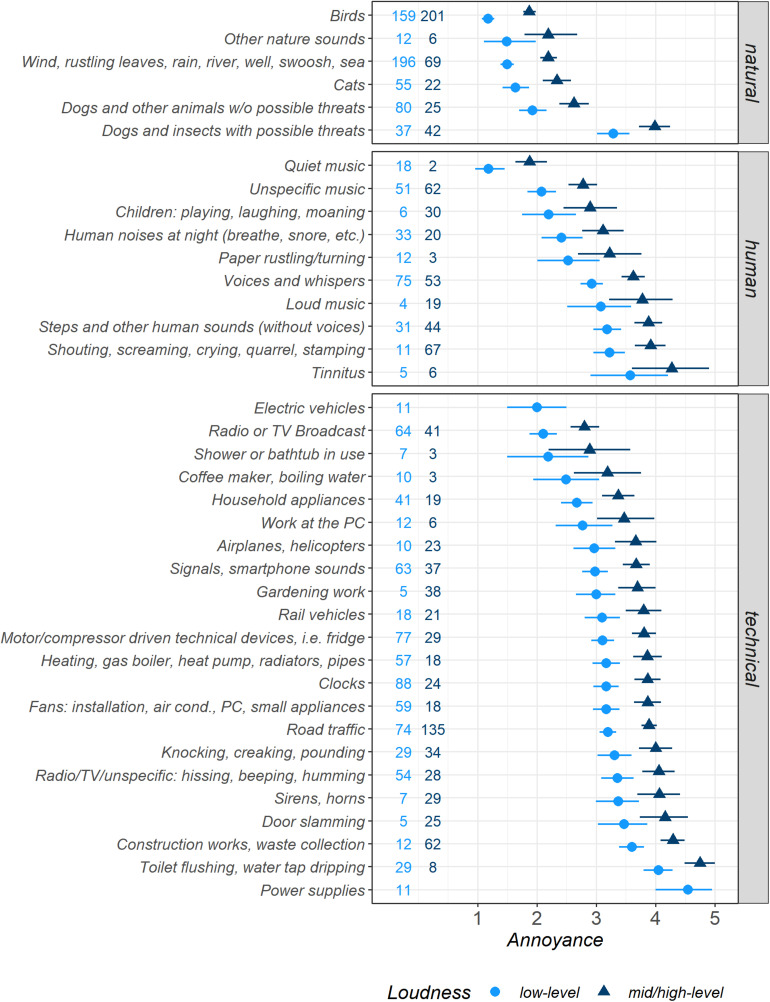
Estimated marginal means for *Annoyance* for sounds from 38 *micro-level sound categories*, separated by binary *Loudness* levels, displayed with 95% confidence intervals (both determined by bootstrapping) and the numbers of observations per category and *Loudness* level subsets. *Very pleasant* = 1, *very annoying* = 5. Model *Micro2*.

**FIGURE A1:**
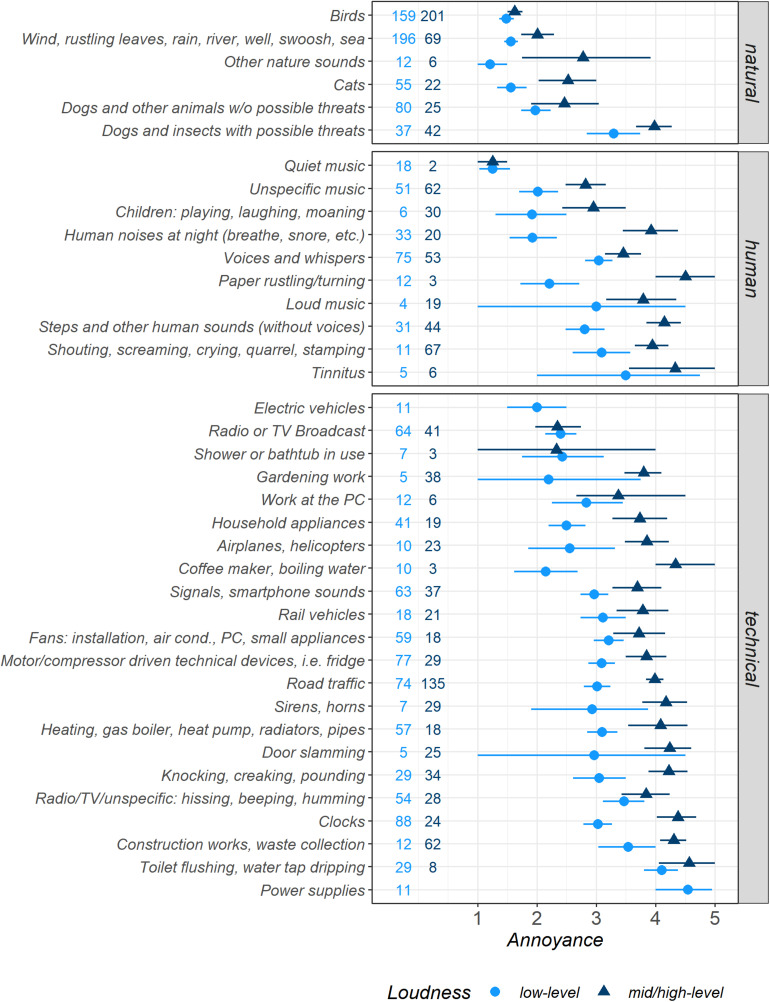
Estimated marginal means for *Annoyance* for sounds from 38 *micro-level sound categories*, separated by binary *Loudness* levels, displayed with 95% confidence intervals (both determined by bootstrapping) and the numbers of observations per category and *Loudness* level subsets. *Very pleasant* = 1, *very annoying* = 5. Models *Micro3a* and *Micro3b*.

Participants reported a total of 2,800 sounds that were merged into 38 *micro-level sound categories* (see Figure 3 with data from model *Micro2*). Similar to the sound categories at the *macro-level* shown in Figure 2, the estimated mean values for the categories at the *micro-level* presented in Figure 3 were equally spaced between the two *Loudness* levels. Since the distributions of the measured data (not shown here) differed for the *micro-level* categories even more than for the *macro-level* categories, the estimated marginal mean values and CIs must be interpreted with the information given above. To provide a more realistic view of these differences, we calculated two different models for both *Loudness* subsets that are not discussed in detail here (models *Micro3*a/b; Figure A1 in the Appendix).

In addition to the *Loudness*-dependent results shown in Figure 3, we now discuss the Loudness-independent estimated marginal means (model *Micro1*). The reports comprised 360 sounds from *Birds*, which constituted the most pleasant natural category (i.e., that with the lowest *Annoyance* ratings), and overall the category with the highest number of reports (*Annoyance M* = 1.56, CI [1.47, 1.66]). With low *Loudness* values, 44% of these sounds were classified as *low-level*. By contrast, *Dogs and insects with possible threats* were the category of natural sounds that participants found most annoying on average (*M* = 3.66, CI [3.38, 3.93]; 47% *low-level* sounds). The remaining natural categories fell close together between the *Birds* value and the *neutral/ambiguous Annoyance* value 3. The second-highest occurrence was reports of noisy, non-tonal sounds like *Wind, rustling leaves, rain, [*…*], sea* (*M* = 1.68, CI [1.56, 1.80]; 74% *low-level*), and *Cats* (*M* = 1.84, CI [1.69, 2.09]; 71% *low-level*), which they perceived as pleasant.

The *human* sound category *Quiet music* was the most pleasant of all *micro-level sound* categories, with 20 reports (*M* = 1.25, CI [1.05 1.50]; 90% *low-level*). The remaining *human* sound categories hovered around the neutral/ambiguous *Annoyance* value 3 between the second most common *human* category *Unspecific music* (*M* = 2.46, CI [2.21, 2.71]; 45% *low-level*; making music, radio play, movie) and the most annoying *human* category *Tinnitus* with 11 reports (*M* = 3.95, CI [3.21, 5.62]; 46% *low-level*). The category *Human noises at night* (*M* = 2.68, CI [2.29, 3.08]; 33% *low-level*) was followed by *Voices and whispers* with the highest number of *human* sounds (*M* = 3.21, CI [3.03, 3.40]; 59% *low-level*).

The most pleasant *technical* category consisted of 11 sounds from *Electric vehicles* (*M* = 2.00, CI [1.50, 2.50]; 100% *low-level*). For *neutral/ambiguous Annoyance* ratings (value 3), there were reports of *Household appliances*, such as washing machines and dishwashers (*M* = 2.89, CI [2.60, 3.20]; 68% *low-level*). Most other *technical* sound categories mainly fell above the *Annoyance* value 3, comprising 112 reports of *Clocks* (*M* = 3.32, CI [3.09, 3.54]; 77% *low-level*) and 106 sounds from motor- or compressor-driven fridges and freezers (*M* = 3.30, CI [3.10, 3.50]; 73% *low-level*). *Road traffic* was the most frequently mentioned *technical* category (*M* = 3.64, CI [3.50, 3.79]; 35% *low-level*).

#### Influence of Location

We evaluated the situation descriptions regarding the categorization of the *Location* in which participants experienced the sound (see Table 1 for the numbers of observations per *Location*) and established six categories: *Garden/Nature*; *Home (indoors)*; *Work/Office*; *Other (indoors)*, such as driving in a car or being in a cinema; *Other (outdoors)*, including walking or riding a bike; and *Undefined*.

**FIGURE 4:**
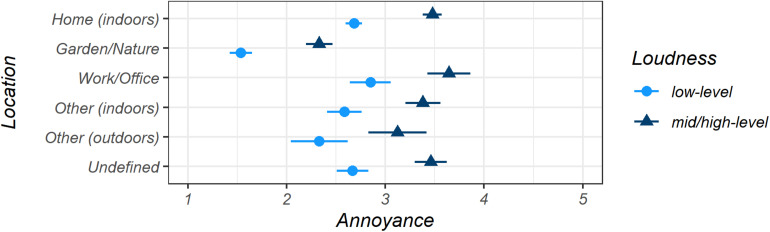
Estimated marginal means for *Annoyance* for all *Location* categories, separated by binary *Loudness* levels, displayed with 95% confidence intervals, both determined by bootstrapping. *Very pleasant* = 1, *very annoying* = 5. Model *Location*.

Figure 4 shows the marginal means for *Annoyance* separated by the binary *Loudness* levels for the six locations given above (model *Location*). *Mid/high-level* sounds were only rated as pleasant for the *Location Garden/Nature* (*M* = 2.33, CI [2.20, 2.47]). In contrast, *mid/high-level* sounds in all other locations were, on average, reported as neutral or slightly annoying (*Home (indoors)*: *M* = 3.48, CI [3.38, 3.57]; *Work/Office*: *M* = 3.64, CI [3.42, 3.86]; *Other (indoors)*: *M* = 3.38, CI [3.20, 3.56]; *Other (outdoors)*: *M* = 3.12, CI [2.83, 3.42]; and *Undefined*: *M* = 3.46, CI [3.30, 3.62]). Unsurprisingly, the average estimated *Annoyance* means for *low-level* sounds from all locations showed less *Annoyance* and were rated from neutral (*Work/Office*: *M* = 2.85, CI [2.64, 3.06]; *Other (indoors*): *M* = 2.59; CI [2.41, 2.76]; *Home (indoors)*: *M* = 2.68, CI [2.60, 2.76]; *Undefined*: *M* = 2.67, CI [2.51, 2.83]) to pleasant (*Other (outdoors)*: *M* = 2.33, CI [2.04, 2.62]), with *Garden/Nature* having the most pleasant ratings on average by far (*M* = 1.54, CI [1.42, 1.65]). The indoor locations and the *Undefined* category showed similar patterns.

Most of the estimated *Annoyance* means for all *Loudness* levels together (not displayed in Figure 4 for better readability) were rated as neutral or ambiguous (*Other (outdoors)*: *M* = 2.61, CI [2.13, 3.20]; *Home (indoors): M* = 3.04, CI [2.96, 3.12; *Work/Office*: *M* = 3.15, CI [2.94, 3.36]; *Other (indoors): M* = 2.92, CI [2.75, 3.08]); *Undefined*: *M* = 3.15, CI [2.98, 3.31]) except for sounds from *Garden/Nature*, which had the only pleasant mean value (*M* = 1.87, CI [1.76, 1.99]).

#### Living Environment

Of all participants, 63.6% stated that they lived in a *calm* or *very calm* area. They reported 63.3% of all assessed sound situations and 64.4% of all *low-level* sounds, as depicted in Table 3. The bootstrapped estimated marginal means of all subsets differed significantly regarding both *Loudness* and *Liveliness* levels, as indicated by their CIs (model *Liveliness*).

**TABLE 3:** Frequencies of observations and estimated marginal means for *Annoyance* judgments, differentiated by the *Liveliness* of the living environment.

	**Lively**				**Calm**				
	**N*_ID_***	**N_obs_**	***M***	**CI**	**N*_ID_***	**N_obs_**	***M***	**CI**	**N_obs sum_**
*Low-level*	474	544	2.62	[2.51, 2.74]	827	984	2.39	[2.30, 2.47]	1,528
*High-level*		484	3.43	[3.31, 3.55]		788	3.20	[3.10, 3.30]	1,272
N_obs sum_		1,028				1,772			2,800

**With bootstrapped confidence intervals for each level of Liveliness of the living environment and each perceived Loudness level. N_ID_ number of participants; N_obs_ number of observations; model Liveliness.**

### Single-Fixed-Factor Models

**FIGURE 5:**
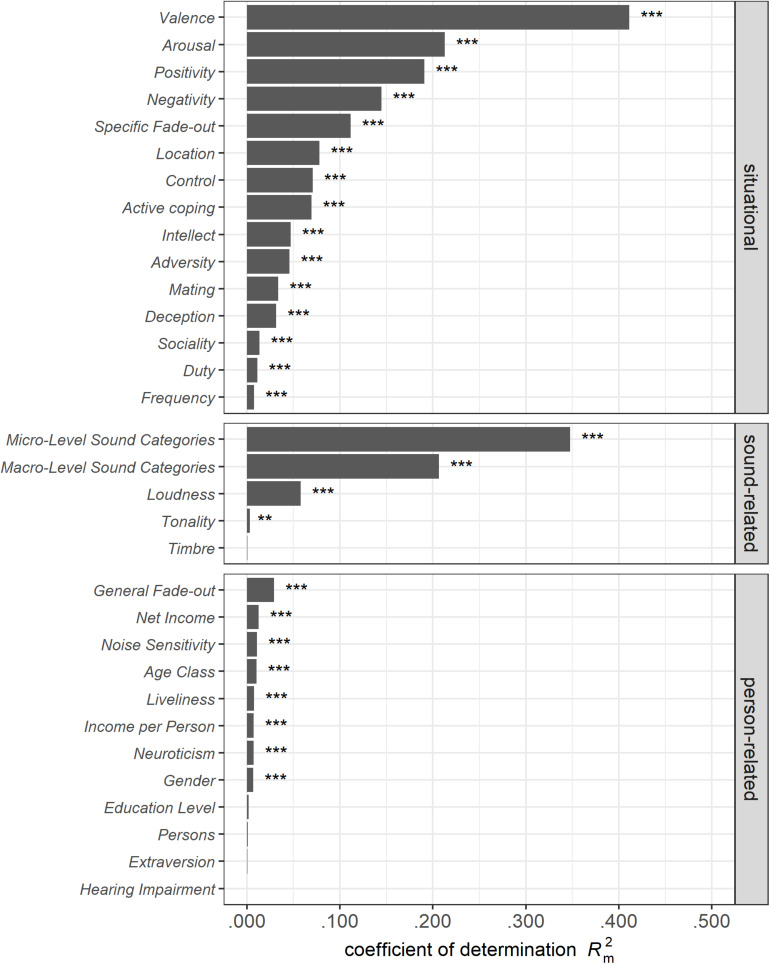
*R*^2^_m_ and probabilities for the assessed variables determined by bivariate analyses of the single-fixed-factor models. Probabilities are given as ^∗∗∗^*p* < 0.001; ^∗∗^*p* < 0.010; ^∗^*p* < 0.050. Model *32SFFA*.

In this section, we present the results of several linear mixed-effects models, each including only one fixed factor, to investigate the effect sizes and directions of the single bivariate relationships related to perceived *Annoyance* of *low-level* sounds (see Table 4, model *32SFF*). Figure 5 (model *32SFFA*) depicts the *R*^2^_m_ and probability values for all variables assessed (except for the soundscape dimensions, which were correlated with our target variable *Annoyance* and thus would lead to tautological findings). Among the crucial variables that explained a substantial amount of variance (own criterion of *R*^2^_m_ ≥ 0.05) were eight situational factors (the affective *Valence*, *Arousal*, *Control*, the *Positivity* and *Negativity* DIAMONDS dimensions, the *Specific Fade-out* ability, the *Location*, and *Active Coping* reaction) and three sound-related factors (*macro-level* and *micro-level sound category* as well as *Loudness*) but no *person-related* factor. These relevant variables will be examined in more detail in the following sections.

**TABLE 4:** *Annoyance* estimates of bivariate single-fixed-factor models including dummy variables of each factor.

	**All *Loudness* levels; factors from single-fixed-factor models**					
**Predictors**	**Estimates**	**[CI]**	***p***	***df***	***R*^2^_m_**	***R*^2^_c_**
**Situational**						
*Valence*	–0.87	−0.91, −0.83	**<0.001**	2717.6	0.411	0.563
*Arousal*	0.63	0.58, 0.68	**<0.001**	2772.3	0.213	0.476
8D: *Positivity*	–0.60	−0.64, −0.55	**<0.001**	2797.1	0.191	0.481
8D: *Negativity*	0.52	0.47, 0.57	**<0.001**	2789.5	0.145	0.438
*Specific Fade-out*	–0.46	−0.51, −0.41	**<0.001**	2795.2	0.112	0.444
*Location*^5^: *Garden/Nature*	–0.38	−0.43, −0.33	**<0.001**	2721.4	0.078	0.431
*Location*^5^: *Work/Office*	0.01	−0.04, 0.06	0.600	2680.7		
*Location*^5^: *Other (indoors)*	–0.01	−0.06, 0.03	0.565	2495.3		
*Location*^5^: *Other (outdoors)*	–0.06	−0.11, −0.02	**0.006**	2623.4		
*Location*^5^: *Undefined*	0.03	−0.02, 0.07	0.279	2740.1		
*Control*	–0.37	−0.42, −0.32	**<0.001**	2797.8	0.071	0.444
*Active Coping*	0.37	0.32, 0.41	**<0.001**	2665.3	0.070	0.461
8D: *Intellect*	–0.30	−0.35, −0.25	**<0.001**	2752.3	0.047	0.434
8D: *Adversity*	0.30	0.24, 0.35	**<0.001**	2781.0	0.046	0.427
8D: *Mating*	–0.25	−0.30, −0.20	**<0.001**	2790.1	0.034	0.435
8D: *Deception*	0.24	0.19, 0.30	**<0.001**	2768.7	0.031	0.420
8D: *Sociality*	–0.16	−0.21, −0.11	**<0.001**	2760.1	0.013	0.422
8D: *Duty*	0.15	0.10, 0.20	**<0.001**	2693.8	0.011	0.415
*Frequency*^7^: less than once a year	0.11	0.06, 0.17	**<0.001**	2724.4	0.008	0.422
*Frequency*^7^: 1.4 times a year	0.06	0.01, 0.11	**0.028**	2628.4		
*Frequency*^7^: 5.11 times a year	0.03	−0.02, 0.09	0.241	2638.1		
*Frequency*^7^: 1.3 times a month	0.07	0.01, 0.13	**0.013**	2546.8		
*Frequency*^7^: 1.3 times a week	0.03	−0.02, 0.09	0.256	2485.0		
*Frequency*^7^: more than once a day	0.02	−0.04, 0.08	0.526	2547.3		
**Sound-related**						
*Macro-level sound category*^8^: *natural*	–0.65	−0.70, −0.61	**<0.001**	2615.9	0.207	0.512
*Macro-level sound category*^8^: *human*	–0.14	−0.18, −0.10	**<0.001**	2461.3		
*Loudness: mid/high-level* (ref.: *low-level*)	0.33	0.28, 0.38	**<0.001**	2767.0	0.058	0.412
*Tonality*	–0.08	−0.12, −0.03	**0.001**	2476.9	0.003	0.426
*Timbre*	–0.03	−0.07, 0.02	0.257	2527.7	<0.001	0.419
**Person-related**						
*General Fade-out*	–0.24	−0.30, −0.18	**<0.001**	1240.4	0.029	0.417
*Net Income* of the household^1^	–0.16	−0.22, −0.09	**<0.001**	1174.1	0.013	0.416
*Noise Sensitivity*	0.14	0.08, 0.21	**<0.001**	1237.2	0.011	0.418
*Liveliness*: *lively* (Ref.: *calm*)	0.12	0.06, 0.19	**<0.001**	1248.7	0.008	0.418
*Neuroticism*	0.12	0.05, 0.18	**<0.001**	1238.8	0.007	0.419
*Income per Person*^1,2^	–0.12	−0.18, −0.05	**<0.001**	1161.6	0.007	0.415
*Gender: male.* (Ref.: *female*)	0.11	0.05, 0.18	**<0.001**	1257.6	0.007	0.419
*Education Class*^4^: ISCED level ≤ 2	0.07	−0.00, 0.14	0.054	1265.96	0.002	0.418
*Education Class*^4^: ISCED level 3	0.02	−0.05, 0.09	0.567	1228.29		
*Persons* living in the household	–0.04	−0.11, 0.02	0.202	1255.8	0.001	0.418
*Extraversion*	0.04	−0.03, 0.10	0.274	1254.6	0.001	0.418
*Age Class*^3^*:* 20 to 40 Years	0.16	0.09, 0.24	**<0.001**	1268.6	0.011	0.419
*Age Class*^3^*:* 41–60 Years	0.07	−0.01, 0.14	0.070	1237.0		
*Hearing Impairment: yes*. (Ref.: *no*)	–0.01	−0.07, 0.06	0.802	1273.4	<0.001	0.418

N*_ID_*	1,301					
N_obs_	2,800					

**^1^N_obs_ 2612, N_ID_ 1215. Reference levels: ^2^ [3200; 4500[EUR, ^3^61–80 years, ^4^ISCED level 4.8, ^5^Home (indoors), ^6^Undefined, ^7^4.7 times a week, ^8^macro-level sound category: technical. N_obs_ number of observations. N_ID_ number of participants, 8D situational eight DIAMONDS. CI confidence intervals and p-values were not corrected. The 38 micro-level sound categories are not shown for better readability. Model 32SFF.* p-values that are significant at the level of α < 0.050 are shown in bold.*

#### Role of Situational Factors

Of the situational variables, *Valence* explained the most variance in the *Annoyance* evaluations (β = −0.87; *R*^2^_m_ = 0.411), followed by *Arousal*, which had a lower but still substantial explanation of variance (β = 0.63; *R*^2^_m_ = 0.213). While positive *Valence* was associated with higher pleasantness (less *Annoyance*), high *Arousal* was related to higher *Annoyance* judgments. Among the situational DIAMONDS dimensions, *Positivity* (β = −0.60; *R*^2^_m_ = 0.191) and *Negativity* (β = 0.52; *R*^2^_m_ = 0.145) revealed the most substantial associations with *Annoyance*. The *Specific Fade-out* ability (β = −0.46; *R*^2^_m_ = 0.112) was followed by several minor effects: Concerning the *Location* (*R*^2^_m_ = 0.078) variable, being in the garden or nature (β = −0.38) instead of staying at home (reference level) was associated with more pleasant sounds. *Active Coping* reactions (β = 0.37; *R*^2^_m_ = 0.070) and perceived *Control* (β = −0.37; *R*^2^_m_ = 0.071) showed similar variance explanations and similar effects but in opposite directions: More *Active Coping* was associated with greater *Annoyance*, while higher levels of perceived *Control* were linked to less annoying sound evaluations. The other situation dimensions, in contrast, revealed *R*^2^_m_ values below 0.050.

#### Role of Sound-Related Factors

Among sound-related variables, both the three *macro-level* (*R*^2^_m_ = 0.207) and 38 *micro*-level sound categories (*R*^2^_m_ = 0.330) explained a substantial amount of variance in the *Annoyance* ratings. Both *natural* (β = −0.65) and *human* (β = −0.14) *macro*-level sound categories had more pleasant sound evaluations compared to the *technical* category. They were followed by perceived *Loudness* (β = 0.33; *R*^2^_m_ = 0.058). Whereas the sound characteristic *Tonality* was significant (β = −0.08; *R*^2^_m_ = 0.003; *p* = 0.001), *Timbre* was not (β = −0.03; *R*^2^_m_ < 0.001; *p* = 0.257). However, both variables showed a negligible effect size (|β| ≤ 0.08; *R*^2^_m_ ≤ 0.003).

#### Role of Person-Related Factors

The *General Fade-out* ability showed the highest, albeit still quite small, variance explanation (*R*^2^_m_ = 0.029) and was negatively associated with *Annoyance* judgments (β = −0.24). The *Net Income* of the household as well as the *Income per Person* revealed very little variance explanation but similar negative effects, suggesting that higher income was associated with more pleasant sound situations. By contrast, the personality traits *Noise Sensitivity* (β = 0.14) and *Neuroticism* (β = 0.12) were significant positive predictors of *Annoyance* judgments but showed minimal *R*^2^_m_ values below 0.010. Moreover, *Extraversion* (β = 0.04) did not show a significant effect at all (*p* = 0.274; *R*^2^_m_ = 0.001). All other demographic variables and the *Liveliness* of the living environment showed minimal *R*^2^_m_ values (<0.010) and/or were insignificant. For *Liveliness* (β = 0.12), a more lively living environment was associated with higher *Annoyance* in sound evaluation. A model for the interaction effect of *Age Class* and *Education Class* on our measured data (model *Age^∗^Edu*, Table 5) confirmed the findings of Miedema and Vos (1999) with these significant influences on annoyance: Younger people (20–40 years) reported less annoying sound situations (β = −0.26) than older people (61–80 years; reference dummy level). Participants with no or up to a lower secondary–level education were slightly less annoyed (β = −0.12) than people with a university-level education (reference dummy level). Finally, only one of the four interactions was significant, showing that young people with an upper secondary–level education experience less annoying sound situations than older people with a university-level education (β = −0.22).

**TABLE 5:** *Annoyance* estimates of the *Age Class* and *Education Class* interaction effect model including all dummy variables of each factor.

	**All *Loudness* levels**					
**Predictors**	**Estimates**	**[CI]**	***p***	***df***	***R*^2^_m_**	***R*^2^_c_**
*Intercept*	2.85	2.79, 2.91	**<0.001**	1257.3	0.016	0.421
*Age Class*^1^*: 20–40 Years*	–0.26	−0.37, −0.15	**<0.001**	1269.3		
*Age Class*^1^*: 41–60 Years*	0.03	−0.08, 0.14	0.601	1245.2		
*Education Class*^2^*: ISCED level* ≤ *2*	–0.12	−0.23, −0.00	**0.042**	1265.6		
*Education Class*^2^*: ISCED level 3*	0.04	−0.07, 0.15	0.504	1248.6		
*Age Class*^1^: 20–40 Years * Education Class^2^*: ISCED level* ≤ *2*	–0.01	−0.21, 0.19	0.925	1274.4		
*Age Class*^1^: 41–60 Years * Education Class^2^*: ISCED level* ≤ *2*	0.01	−0.18, 0.20	0.922	1256.5		
*Age Class*^1^: 20–40 Years * Education Class^2^*: ISCED level 3*	–0.22	−0.41, −0.03	**0.025**	1264.0		
*Age Class*^1^: 41–60 Years * Education Class^2^*: ISCED level 3*	–0.01	−0.20, 0.18	0.886	1233.4		

N*_ID_*	1,301					
N_obs_	2,800					

**Reference levels: ^1^61–80 years, ^2^ISCED level 4.8. N_obs_ number of observations. N_ID_ number of participants, CI bootstrapped confidence intervals were given at α = 0.05. Model Age^∗^Edu.* p-values that are significant at the level of α < 0.050 are shown in bold.*

### Comprehensive Models

In this section, we present the comprehensive model *CML1* predicting *Annoyance* ratings derived from the Lasso regression method for variable selection. Some variables had not been processed due to missing values (*Net Income* and *Income per Person*)—which were not allowed for the regularization method—or having too many factor levels (*Micro Sound Categories*). The minimum cross-validation error (*CV* = 0.81) was reached at a tuning parameter value of λ _opt_ = 96.0 (see dashed line in Figure 1). This optimal compromise between a model in which all our variables were retained as contributors and a model without any fixed factors left over (at a λ _max_ = 2,256) incorporated the most important variables shown in Table 6. This prediction model explained over half of the variance of the *Annoyance* evaluations (*R*^2^_m_ = 0.570).

**TABLE 6:** Estimations of Lasso-selected parameters for the full dataset and two *Loudness* subsets.

	**All levels**		**Low-level**		**Mid/high-level**	
λ_opt_	96.0		90.8		117.0	
λ_max_	2,256		1,231		1,124	
*CV*	0.81		0.72		0.93	

**Predictors**	**Estimates**	***p***	**Estimates**	***p***	**Estimates**	***p***

	**[CI]**	***df***	**[CI]**	***df***	**[CI]**	***df***
(Intercept)	2.84	**<0.001**	2.47	**<0.001**	3.27	**<0.001**
	2.80, 2.88	1193.8	2.42, 2.51	833.9	3.22, 3.33	735.1
**Situational**						
*Valence*	−0.47	**<0.001**	−0.49	**<0.001**	−0.43	**<0.001**
	−0.51, −0.42	2787.7	−0.54, −0.43	1517.5	−0.49, −0.36	1252.0
*Arousal*	0.13	**<0.001**	0.14	**<0.001**	0.11	**0.001**
	0.09, 0.18	2769.5	0.09, 0.19	1472.8	0.05, 0.18	1259.8
*Positivity*	−0.19	**<0.001**	−0.13	**<0.001**	−0.24	**<0.001**
	−0.22, −0.15	2778.9	−0.18, −0.08	1506.6	−0.30, −0.18	1263.9
*Specific Fade*−*out*	−0.20	**<0.001**	−0.14	**<0.001**	−0.23	**<0.001**
	−0.24, −0.16	2788.6	−0.19, −0.10	1508.0	−0.29, −0.17	1262.4
*Active Coping*	0.13	**<0.001**	0.11	**<0.001**	0.15	**<0.001**
	0.09, 0.16	2785.4	0.07, 0.16	1509.6	0.10, 0.20	1257.5
**Sound-related**						
*Natural* sounds	−0.38	**<0.001**	−0.35	**<0.001**	−0.43	**<0.001**
	−0.41, −0.34	2693.2	−0.40, −0.31	1496.3	−0.49, −0.37	1229.7
*Human* sounds	−0.15	**<0.001**	−0.17	**<0.001**	−0.14	**<0.001**
	−0.18, −0.11	2635.1	−0.21, −0.12	1455.7	−0.19, −0.08	1173.2
*Mid/high-level*	0.17	**<0.001**	(grouping variable)		(grouping variable)	
	0.14, 0.21	2787.3				
*Tonality*	−0.05	**0.002**				
	−0.09, −0.02	2700.9				
**Person-related**						
*General Fade-out*	−0.01	0.526				
	−0.05, 0.03	1325.9				
**Random effects**						
σ^2^	0.60		0.55		0.65	
τ_00_	0.21*_ID_*		0.16*_ID_*		0.26*_ID_*	
ICC_adj_	0.26		0.22		0.29	
N*_ID_*	1,301*_ID_*		930*_ID_*		798*_ID_*	

N_obs_	2,800_obs_		1,528_obs_		1,272_obs_	

Marginal *R*^2^	0.570		0.532		0.543	
Conditional *R*^2^	0.680		0.637		0.674	

**Reference micro-level sound category: technical. Reference Loudness: low-level. λ_max_ highest value of Lasso tuning parameter, λ_opt_ optimal value of Lasso tuning parameter, CV cross-validation error, i.e., the mean of the mean square errors of all cross-validation folds, σ^2^ residual (within-subject) variance, τ_00_ random intercept (between-subject) variance (i.e., variation between individual intercepts and average intercept), ICC_adj_*, *adjusted intraclass-correlation coefficient = τ_00_/(τ_00_ + σ^2^) describes the variance—including the fixed-effects variance—between participants; N _ID_, number of persons; N_obs_*, *number of observations; CI, confidence intervals were given at α = 0.05. Models CML1/2/3. p-values that are significant at the level of α< 0.050 are shown in bold.**

The most crucial fixed factor was the affect *Valence* (β = −0.47, CI [−0.51, −0.42]) followed by the *natural* sound category (β = −0.38, CI [−0.41, −0.34]) and the *Specific Fade-out* ability (β = −0.20, CI [−0.24, −0.16]). The situational variable *Positivity* (β = −0.19, CI [−0.22, −0.15]) was included in the model, whereas *Negativity* was excluded. Lower *Annoyance* ratings were therefore related to higher (more positive) *Valence* scores, *natural* sounds as opposed to *technical* ones, and the stronger ability of respondents to fade out sounds. In contrast to the aforementioned negative effects, the fifth most important variable was the positive effect *mid/high-level Loudness* (β = 0.17, CI [0.14, 0.21]), followed by the positive effects *Active Coping* (β = 0.13, CI [0.09, 0.16]) and *Arousal* (β = 0.13, CI [0.09, 0.18]). That is, higher *Annoyance* values were related to higher *Loudness*, higher *Arousal*, and higher *Active Coping* scores. The sound characteristic *Tonality* had the smallest significant effect (β = −0.05, CI [−0.09, −0.02]). Finally, the *General Fade-out* capability (β = −0.01, CI [−0.05, 0.03]) was included as the only variable with a non-significant *p*-value (*p* = 0.526), whereas all other effects had highly significant *p*-values (*p* < 0.010).

Concerning the random effect *ID*, the residual (within-subject) variance σ^2^ = 0.60 and the random intercept (between-subject) variance τ_00_ = 0.21 were observed. The quite high within-subject variance of the *Annoyance* ratings may be due to high variation in the characteristics of the sounds and situations reported by participants. This variation is slightly smaller for the subset of sounds reported as low-level (σ^2^ = 0.55). An interpretation of this might be that the low-level sounds reported by participants were generally less annoying, whereas mid/high-level sounds (σ^2^ = 0.65) can be very annoying or even very pleasant—for example, imagine playing your favorite music or the *Bird* sounds that were reported as being *mid/high-level*. This can be seen in Figure 2, which shows the distribution for the two sound level subsets (additionally separated by *macro-level sound category*, model *Macro2*). A similar relationship between the sound level subsets can be observed for the between-subject variation τ_00_ of the random effect. These values were 0.3–0.4 times the within-subject variances. Unsurprisingly, the relationship between the sound level subsets mentioned above can also be found in the standard deviations of the raw data (*SD*_all_ = 1.39; *SD*_low__–__level_ = 1.24; *SD*_mid/__high__–__level_ = 1.42; see also the distributions of the reported raw-data in Figure 2). Finally, an adjusted (i.e., conditional) intraclass-correlation coefficient for the full dataset—ICC_adj_ = τ_00_/(τ_00_ + σ^2^) = 0.26—described the proportion of explained variance to total variance (including the fixed effects) due to differences between participants which were represented by the random effect *ID*. From a critical perspective, all of the above differences in variances and their interpretation may be strongly influenced by the huge variety in the sounds reported by participants due to the fact that each participant reported individual sounds, as no audio was played back and no grouped listening (as in sound walks) was performed.

In addition to the aforementioned model computed over the full dataset, we derived two further models using the Lasso regression method for the subsets of *low-level* (model *CML2*) versus *mid/high-level* (model *CML3*) observations. This was done to investigate whether evaluations of both *low-* and *mid/high-level* sounds would follow similar patterns. Both models showed similar but slightly smaller marginal *R*^2^-values (*R*^2^_m_____low__–__level_ = 0.532; *R*^2^_m_____mid/__high__–__level_ = 0.543) compared to the overall model. The *Loudness* variable was no longer included in the sub-models, presumably because it served as the grouping variable. The two variables *Tonality* and *General Fade-out* were also excluded by the Lasso regression at the optimal tuning parameters (*low-level*: λ _opt_ = 90.8, λ _max_ = 1,231; *mid/high-level*: λ_opt_ = 117.0, λ _max_ = 1,124). Compared to the overall model, the model for the *low-level* subset showed a slightly smaller cross-validation error (*CV* = 0.72). In contrast, the error of the *mid/high-level* model was slightly higher (*CV* = 0.93). When comparing the predictor estimates of the two models based on their CIs, no significant differences were observed, and both showed the same selected variables.

A model (*CMSFF1*) containing all variables that were significant in the bivariate analyses (subsections of section “Design and Questionnaire” and Figure 5) and showed an *R*^2^_m_ ≥ 0.050 is shown in Table 7. The variables selected in this way confirmed the variable selection by the Lasso regularization method. The variable *Control*, which was meaningful in the bivariate analysis (β = −0.37; *R*^2^_m_ = 0.071; *p* < 0.001), became unimportant in the comprehensive model (β = 0.00; *p* = 0.910). Some *Location* levels were inconsistently significant within each *Loudness* subset as well as between subsets. Although the Lasso variable selection method—in a misleading manner—selected *General Fade-out*, which was not significant, no *person-related* variable we assessed achieved an *R*^2^_m_ of 0.050 in the single-fixed-factor models, and such variables were therefore excluded in this comprehensive linear model.

**TABLE 7:** Comprehensive model of all parameters from the bivariate analyses that reached an *R*^2^_m_ ≥ 0.050, respectively; with the full dataset and two *Loudness* subsets.

	**All levels**		**Low-level**		**Mid/high-level**	
***Predictors***	***Estimates***	***p***	***Estimates***	***p***	***Estimates***	***p***
	**[CI]**	***df***	**[CI]**	***df***	**[CI]**	***df***
(Intercept)	2.84	**<0.001**	2.47	**<0.001**	3.27	**<0.001**
	2.80, 2.88	1188.3	2.42, 2.51	820.2	3.22, 3.33	722.1
**Situational**						
*Valence*	−0.46	**<0.001**	−0.47	**<0.001**	−0.42	**<0.001**
	−0.51, −0.41	2779.7	−0.53, −0.42	1510.9	−0.49, −0.35	1231.2
*Arousal*	0.13	**<0.001**	0.12	**<0.001**	0.11	**0.001**
	0.08, 0.17	2770.8	0.07, 0.18	1469.9	0.04, 0.17	1254.8
*Positivity*	−0.17	**<0.001**	−0.12	**<0.001**	−0.23	**<0.001**
	−0.21, −0.13	2768.3	−0.17, −0.06	1489.0	−0.30, −0.17	1256.7
*Negativity*	0.06	0.007	0.06	0.013	0.04	0.173
	0.02, 0.10	2778.5	0.01, 0.11	1502.4	−0.02, 0.11	1254.7
*Specific Fade-out*	−0.19	**<0.001**	−0.14	**<0.001**	−0.23	**<0.001**
	−0.23, −0.16	2772.0	−0.19, −0.10	1497.9	−0.29, −0.17	1255.0
*Location: Garden/Nature*	−0.02	0.299	−0.03	0.333	−0.03	0.398
	−0.06, 0.02	2762.6	−0.08, 0.03	1511.4	−0.09, 0.03	1256.4
*Location: Work/Office*	0.00	0.840	0.02	0.298	−0.03	0.276
	−0.04, 0.03	2771.0	−0.02, 0.07	1512.2	−0.08, 0.02	1226.9
*Location: Other (indoors)*	−0.05	**0.003**	−0.02	0.370	−0.09	**0.001**
	−0.08, −0.02	2659.1	−0.06, 0.02	1427.7	−0.14, −0.04	1200.9
*Location: Other (outdoors)*	−0.03	0.052	−0.06	**0.005**	0.02	0.515
	−0.07, 0.00	2738.1	−0.10, −0.02	1494.9	−0.03, 0.07	1228.0
*Location: Undefined*	−0.03	0.126	0.01	0.680	−0.07	**0.013**
	−0.06, 0.01	2782.2	−0.03, 0.05	1502.3	−0.12, −0.01	1255.1
*Control*	0.00	0.910	−0.01	0.758	0.02	0.442
	−0.04, 0.04	2736.7	−0.06, 0.04	1453.5	−0.04, 0.08	1257.0
*Active Coping*	0.12	**<0.001**	0.10	**<0.001**	0.14	**<0.001**
	0.09, 0.16	2781.1	0.06, 0.15	1505.4	0.08, 0.19	1249.9
**Sound-related**						
*Natural* sounds	−0.38	**<0.001**	−0.35	**<0.001**	−0.44	**<0.001**
	−0.42, −0.34	2652.8	−0.40, −0.29	1487.9	−0.50, −0.37	1210.1
*Human* sounds	−0.15	**<0.001**	−0.17	**<0.001**	−0.13	**<0.001**
	−0.18, −0.11	2636.7	−0.21, −0.12	1455.9	−0.19, −0.08	1168.0
*Mid/high-level*	0.16	**<0.001**	(Grouping variable)		(Grouping variable)	
	0.12, 0.19	2781.7				
**Random effects**						
σ^2^	0.61		0.56		0.64	
τ_00_	0.20*_ID_*		0.15*_ID_*		0.26*_ID_*	
ICC	0.25		0.21		0.29	
N*_ID_*	1,301*_ID_*		930*_ID_*		798*_ID_*	

N_obs_	2,800_obs_		1,528_obs_		1,272_obs_	

Marginal *R*^2^	0.573		0.538		0.549	
Conditional *R*^2^	0.678		0.635		0.679	

**Reference Location: Home (indoors). Reference micro-level sound category: technical. Reference Loudness: low-level. σ^2^*, *residual (within-subject) variance; τ_00_*, *random intercept (between-subject) variance; ICC, intraclass-correlation coefficient; N _ID_*, *number of persons; N_obs_*, *number of observations; CI, confidence intervals. Models CMSFF1/2/3. p-values that are significant at the level of α < 0.050 are shown in bold.**

## Discussion

### Summary

In this online study, we investigated the human perception of low-level environmental sounds and the influencing effects of sound-related, situational, and person-related factors. Moreover, we investigated whether variable-selection methods from linear machine-learning algorithms can aid noise effects and soundscape research by creating comprehensive models which can reliably predict and explain a considerable amount of variance in unseen data which was not used in the training when the model was built.

The results of our study corroborate previous findings suggesting that sound evaluations are dependent on myriad influencing factors, in particular situational factors (Fields, 1993; Wolsink et al., 1993; Stallen, 1999; Job et al., 2007; Kroesen et al., 2008; Steffens et al., 2017). Moreover, we demonstrated that linear mixed-effects models combined with novel machine learning variable-selection techniques are applicable in hypothesis testing in noise effects and soundscape research. Furthermore, they can overcome problems associated with overfitting and multicollinearity when many situational and person-related variables are included in the course of a multiple regression. The feasibility of these techniques is further supported by our extensive and time-consuming bivariate analyses of the single variables, which overall led to similar results.

To the best of our knowledge, this is the first study in the realm of sound perception that takes into account such a large number of psychological variables and utilizes linear machine learning to overcome the aforementioned statistical problems. In addition, the established models derived from the percentile-Lasso method maintain interpretability due to the linear, additive effects of the predictor variables on the outcome variable, as opposed to widespread deep learning approaches that obfuscate those relationships. The percentile-Lasso regression approach is assumed to be particularly useful if multiple (psycho-)acoustic parameters—usually highly correlated—are also taken into account in the course of more comprehensive future studies and models. Moreover, the combination of multilevel modeling and the percentile-Lasso approach will also allow time-series analyses and the separate modeling of inter- and intra-individual processes relevant to everyday sound perception.

The more detailed analysis of our data further supported the feasibility of using the three most frequently reported macro-level sound categories (natural, human, and technical; Axelsson et al., 2010; Bones et al., 2018; ISO/TS 12913-2,ISO, 2018), whose mean annoyance ratings differed significantly. In addition, we derived 38 micro-level sound categories from sound and situation descriptions, which shed light on the kinds of sounds people experience in their day-to-day life, including their prevalence and how they were evaluated depending on the loudness level.

Since the bootstrapping for the marginal mean values is based on resampling through the level 2 cluster variable, it cannot reproduce well the different distributions of, e.g., *low-level* sounds and *mid/high-level* sounds. When a second fixed factor was included in the models, the differences between the marginal means of the two loudness levels were equal across all levels of the other fixed factor (see Figures 2–4). For study designs with multiple fixed factor and non-homogeneously distributed data, a specific statistical approach must be developed in future research that relates the clustering to several levels of multiple factors.

Regarding the evaluation of low-level sounds, our models revealed the expected significant positive effects of perceived loudness on annoyance perception. However, the effect size of loudness was oftentimes smaller than that of non-auditory variables (Job, 1996; Kim et al., 2017; World Health Organization, 2018), which might be a result of the study design’s focusing on low-level sounds. A significant loudness-dependent difference in annoyance mean values was indeed observed for the three macro-level sound categories (natural, human, and technical). However, none of the effects of the Lasso-selected variables in the optimal models for different loudness levels differed significantly. As such, human sound perception strategies seem to be loudness-level independent, as the same predictors for the two sub-models (low-level and mid/high-level sounds) were selected by the Lasso. This could be affected by the fact that no acoustic stimuli were presented. The assessment of sounds that are remembered after days or weeks may be affected by a memory bias. An example could be the Peak-End rule, according to which people usually base their retrospective judgments on the most intense (Peak) and the last event (End) (Steffens et al., 2017). The inclusion of more cognitive processing may further bias the assessment compared to an *in situ* evaluation. Another explanation might be that some participants remembered a low-level sound while answering the questionnaire but—because no sound was provided—connected it with the sound source that they might have experienced in other situations at a shorter distance, i.e., higher loudness. Such a justification could also explain why the other sound characteristics—tonality and timbre—contributed negligibly to explaining the variance of annoyance or were even not significant. Non-significant predictors, like the *General Fade-out*, may be present in the model, especially resulting from the usage of the 1-SE rule. The reason for this lies in the Lasso variable selection method: The Lasso excludes predictors based on regularization but not *p*-values. The model selection is based on cross-validated MSE values in combination with the 1-SE rule to detect the most generalized and parsimonious model with low prediction error. Finally, we performed statistical testing and *p*-value analysis after the model selection process.

Our results of the bivariate analyses of the DIAMONDS psychological situation dimensions (Rauthmann et al., 2014) showed only two strong associations. As expected, positive situations were associated with low annoyance (i.e., pleasant) judgments and negative situations with high annoyance. The other six dimensions showed only small effects; sociality and duty were insignificant. A situation with intellectual, romantic, or social aspects was associated with more pleasant sounds, whereas adversity, deception, and duty were connected with greater annoyance. A reason for the relatively low contribution of the DIAMONDS dimensions to the annoyance perception may be that all participants rated individual sound situations and incorporated high diversity in objective environmental characteristics that were assessed—in the worst case—only once. The situational variables then become individual perceptions, i.e., personal variables (Rauthmann and Sherman, 2020). This represents a particular challenge for online studies and more valid field studies that must capture situations in a way that reduces otherwise enormous diversity.

Another situational variable—perceived control, often interpreted as perceived dominance over the situation—showed a minor effect in our analyses. In contrast, other studies have emphasized control as an important—if not essential—non-auditory factor with a negative effect on annoyance (Kjellberg et al., 1996; Stallen, 1999; Kroesen et al., 2008). An explanation might be found in the retrospective study design, as participants may not have been able to remember every aspect of the situation described, leading to bias. Nevertheless, our results are still consistent with the findings of Graeven (1975), who reported a small but significant effect of control over noise in the neighborhood or at home, and with the more recent results of Hatfield et al. (2002), who found a small negative effect of perceived control on sleep, reading disturbance, and general symptoms.

Although other studies have discussed coping as one of the top three non-acoustical factors of sound perception (Stallen, 1999; Kroesen et al., 2008), active coping was a minor situational factor for predicting annoyance in our study, both in the bivariate and the Lasso regression model. More active coping was associated with greater annoyance. Our result is in line with several studies positing that coping can be seen as a consequence of annoyance (Glass et al., 1972; Botteldooren and Lercher, 2004; Park et al., 2016). At first glance, we observed a direction of the coping effect contrary to that reported by Kroesen et al. (2008). It seems plausible that one might only feel the strong need for coping activity if one feels highly annoyed by a given situation. Kroesen et al., however, defined a different aspect of coping, namely “coping capacity,” which diminishes if one’s ability to face a threat is limited or reduced. As a consequence of being not able to cope with the situation, stress rises. By extension, perceiving a higher coping capacity leads to less annoyance.

In our study, the self-determined ability to fade out the specific reported sound in the specific situation was a crucial factor in explaining the variance of annoyance after valence, arousal, positivity, and (though not in the Lasso-selected variables) negativity. This is quite interesting, as to our knowledge there is no research on this topic available. As our study was very broad in scope, we could not explore every aspect in depth. It seems worthwhile to further study this construct, its antecedents and consequences, and its person-related (e.g., attention deficit disorder), situational (e.g., fatigue), and sound-related (e.g., saliency) correlates. Here, it would be particularly interesting to investigate the stability and situation dependency of this ability and whether its effect on annoyance evaluation can be reproduced in other contexts, such as the field or the laboratory.

Interestingly, noise sensitivity showed a minimal variance explanation with a small positive effect, meaning that higher noise sensitivity was associated with greater annoyance. Other studies have identified noise sensitivity as the most crucial factor in the annoyance responses caused by noise, considerably stronger than the exposure level (Job, 1988; Öhrström et al., 1988; Ryu and Jeon, 2011). In contrast, Stölzel (2004) and Kroesen et al. (2008) found no added explained variance or only a small correlation between noise sensitivity and annoyance, which is in line with our findings. One explanation for this discrepancy could be that we used a short questionnaire, the LEF-K (Zimmer and Ellermeier, 1998). Another could be that, according to other researchers, noise sensitivity may be seen as a multidimensional construct, which means that it might be different for various aspects of daily life (Schütte et al., 2007) and sound levels (Job, 1999). Therefore, an (additional) measurement such as the NoiSeQ (Schütte et al., 2007) or the short version NoiSeQ-R, which considers three everyday scenarios (Griefahn, 2008), might be advisable for future studies.

Since 1260 (45%) of all 2800 reported sounds were rated as mid/high-level—although we were interested in the low-level sounds perceived by the participants—one could hypothesize that some participants indicated the presumed volume of the sound source rather than what they heard. It could also be the case that when asked to report low-level sounds, the participants intuitively thought of a low-level sound, such as birdsong. Later, when asked to evaluate the loudness, they probably made a more cognitive evaluation, perhaps putting the sound into a context or comparing it with other sounds and situations. For example, birdsong might appear loud in a quiet morning, while it is certainly still a low-level sound compared to an accelerating bus passing by.

### Limitations

Some limitations associated with the test design should be addressed. First, we provided no acoustic stimuli to participants. Instead, they recalled sounds, situations, and behavior, potentially introducing a memory bias (Steffens et al., 2017; Greb et al., 2019). Notwithstanding, our results (for example, in terms of valence and arousal) revealed similar values compared to studies that used acoustical stimuli (e.g., Hall et al., 2013). Furthermore, the data we assessed allowed for the interpretation of correlative relationships between variables but revealed neither directions of effects nor moderation and mediation effects. In addition, each participant reported only one to three observations, which makes it inappropriate to calculate personal means of otherwise time-varying measures. All of these drawbacks can be addressed by conducting a field study applying the experience sampling method and by obtaining a high number of repeated measures for each participant. The authors are preparing a large-scale field study, including on-site sound recordings, which aims to replicate and extend the findings of this study.

## Conclusion

Despite the limitations mentioned above, our study shows how to deal with many influencing variables in the field of sound perception using machine learning for the selection of the most essential variables. Even though no actual acoustical stimuli were used, our recall-based online study revealed some crucial factors associated with annoyance judgments (valence, arousal, sound categories, and mental fade-out ability). The results of this study also have practical implications for manufacturers of technical equipment and domestic installations, as even low-level sounds—such as toilet flushing, which was associated with high annoyance ratings in our study—can be prominent (Kuwano et al., 2003). Manufacturers of heating installations, for example, may offer their customers a sense of perceived control that can lower annoyance perceptions by enabling customers to adjust the flow rate of the heating installation to reduce flow noise, if temporarily desired.

## Data Availability Statement

The data sets presented in this article are not readily available as supplementary material, as the researchers plan to conduct further analyses. Requests for access to the data sets can be sent to SV, siegbert.versuemer@hs-duesseldorf.de.

## Ethics Statement

The studies involving human participants were reviewed and approved by the Ethics Committee of the Medical Faculty of the University of Duisburg-Essen, Germany. All participants provided digital written informed consent by confirming the declaration on data collection and processing before participating. They were anonymous to the researchers at all times.

## Author Contributions

SV and JS designed the study, interpreted data, reviewed, and edited the manuscript. SV collected the data, performed the statistical analysis, programmed the diagrams, and wrote the first draft of the manuscript. PB implemented machine learning, wrote the corresponding passage in the “Materials and Methods” section, and consulted on statistics during editing. JB-S provided initial concept and consulted in the evaluation process. All authors approved the final version of the manuscript.

## Conflict of Interest

The authors declare that the research was conducted in the absence of any commercial or financial relationships that could be construed as a potential conflict of interest.

## Footnotes

1 https://www.respondi.com/

2 https://www.limesurvey.org/

3 Instead of “1” and “a,” the sound and situation descriptions given by the participants were inserted here.

4 *N* (number of subjects with two or more ratings) = 3,039; *n* (number of ratings) = 2; *k* (number of categories) = 38 + 2.

5 *N* (number of subjects with two or more ratings) = 3,039; *n* (number of ratings) = 2; *k* (number of categories) = 3 + 2; *B* (number of bootstrap samples) = 1,000.

6 *N* (number of subjects with two or more ratings) = 2,778; *n* (number of ratings) = 2; *k* (number of categories) = 6; *B* (number of bootstrap samples) = 1,000. The *N* here is smaller than the *N* for the sound categories because locations have not been clustered for nonsensical reports.

7 https://easystats.github.io/performance/

8 SE, Standard Error.
