# Supplementary material for: Modeling Evaluations of Low-Level Sounds in Everyday Situations Using Linear Machine Learning for Variable Selection
Source: Front Psychol. 2020 Oct 23;11:570761. doi: 10.3389/fpsyg.2020.570761 (PMC7644977; doi:10.3389/fpsyg.2020.570761)
Supplement: Supplementary file 1 [file Data_Sheet_1.PDF]

Modeling evaluations of low-level sounds in everyday situations using linear machine learning for variable selection

Supplemental Material:

Online Questionnaire

The original texts and screenshots from the questionnaire are presented here. Screenshots were taken from the open-source online survey platform LimeSurvey[1]. In addition to the screenshots, details of the question or task, further information, scale levels, the item used in the analyses, and references were provided as required.

Content

1. Online questionnaire 2
2. References 21

[1] https://www.limesurvey.org/en/;
creative-commons licence CC BY-SA 3.0; https://creativecommons.org/licenses/by-sa/3.0/

1. Online questionnaire

Sehr geehrte Teilnehmerinnen und Teilnehmer,<br>vielen Dank für Ihr Interesse an dieser Umfrage. Sie findet im Rahmen eines vom Bundeministeriums für Bildung und Forschung geförderten Forschungsprojektes am Insstitute of Sound and Vibration Engineering (ISAVE) an der Hochschule Düsseldorf statt. Die Umfrage dient der Erhebung zum einen von Situationen, in denen Sie leise Geräusche wahrnehmen, zum anderen Ihrer subjektiven Bewertung dieser Geräusche.<br>Wir empfehlen, die Umfrage auf einem großen Display z. B. auf einem Rechner oder einem Tablet durchzuführen.<br>Alle Ihre Angaben werden streng vertraulich behandelt. Der Datensatz wird vom ISAVE ("Institute of Sound and Vibration Engineering") der Hochschule Düsseldorf nach den geltenden Regeln erhoben und verarbeitet. Für die Darstellung der Ergebnisse im Rahmen etwaiger Veröffentlichungen werden die Daten aller Teilnehmer/innen aggregiert. Das heißt, dass keine Rückschlüsse von den Ergebnissen auf einzelne Teilnehmer/innen möglich sind. Wir speichern keine IPAdressen, wodurch wir nicht auf Ihre Person schließen können.<br><br>Bei Fragen wenden Sie sich bitte an .....@hs-duesseldorf.de.<br><br>Vielen Dank!
Dear participants, thank you very much for your interest in this survey. It is part of a research project sponsored by the German Federal Ministry of Education and Research at the Institute of Sound and Vibration Engineering (ISAVE) at the University of Applied Sciences Düsseldorf.<br><br>The survey is designed to collect information about situations in which you perceive soft sounds and your subjective evaluation of these sounds.<br><br>We recommend completing the survey on a large display such as a computer or tablet.<br><br>All your information will be treated in strict confidence. The data set will be collected and processed by ISAVE ("Institute of Sound and Vibration Engineering") of the University of Applied Sciences Düsseldorf according to the applicable rules. The data of all participants will be aggregated for the presentation of the results in any publications. Thus, no conclusions can be drawn from the results to individual participants. We do not store IP addresses, which means that we cannot identify you personally.<br><br>If you have any questions, please contact .....@hs-duesseldorf.de.<br><br>Thanks a lot!<br><br>To participate in the survey, please accept our privacy policy.

*Geben Sie Ihr Alter ein.

In dieses Feld dürfen nur Zahlen eingegeben werden.

Question/Task	Enter your age.
Information	Only numbers may be entered in this field.
Scale Levels	Numerical variable. No levels.
Item *Age*	

*Geben Sie bitte Ihr Geschlecht an.

Bitte wählen Sie eine der folgenden Antworten:

- männlich
- weiblich
- divers

Question/Task	Please enter your sex.
Information	Please choose one of the following answers:
Scale Levels	male; female; diverse
Item *Gender*	

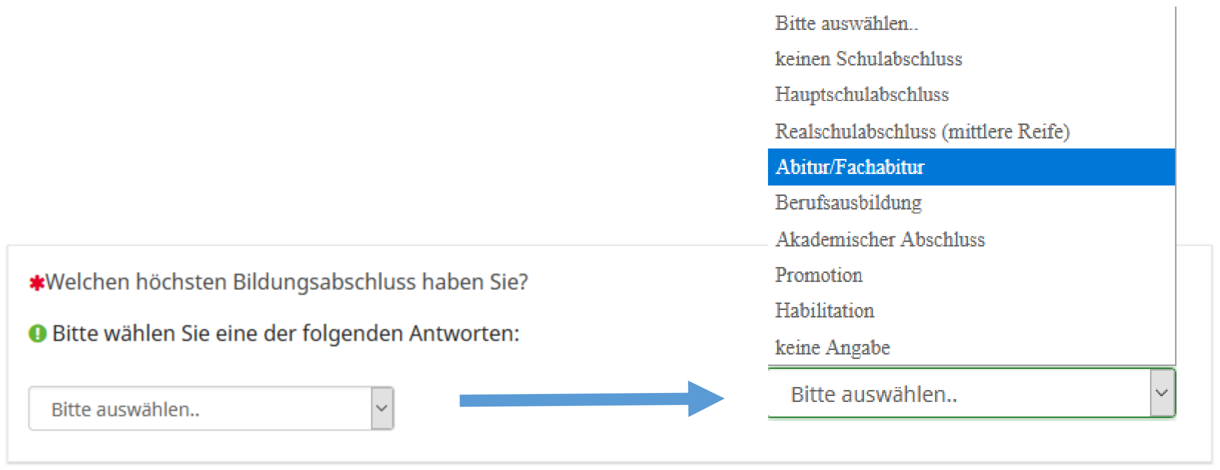

Question/Task	What is your highest educational level?
Information	Please choose one of the following answers:
Scale Levels	no school-leaving qualification<br>primary school<br>lower secondary school<br>upper secondary school<br>vocational education<br>academic degree<br>doctoral level<br>habilitation level
Item *Education Level*	
References	UNESCO Institute for Statistics (2015);<br>Schneider (2008)

*Bitte geben Sie ihre Nationalität an.

Bitte wählen Sie eine der folgenden Antworten:

- deutsch
- andere

Question/Task	Please enter your nationality.
Information	Please choose one of the following answers:
Scale Levels	German; other.
Item *Nationality*	

*Bitte geben Sie an, wie viele Personen (Sie eingeschlossen) in ihrem Haushalt leben.

Bitte wählen Sie eine der folgenden Antworten:

- 1
- 2
- 3
- 4
- 5
- 6 und mehr

Question/Task	Please indicate how many people (including you) live in your household.
Information	Please choose one of the following answers:
Scale Levels	1; 2; 3; 4; 5; 6 and more.
Item *Persons*	

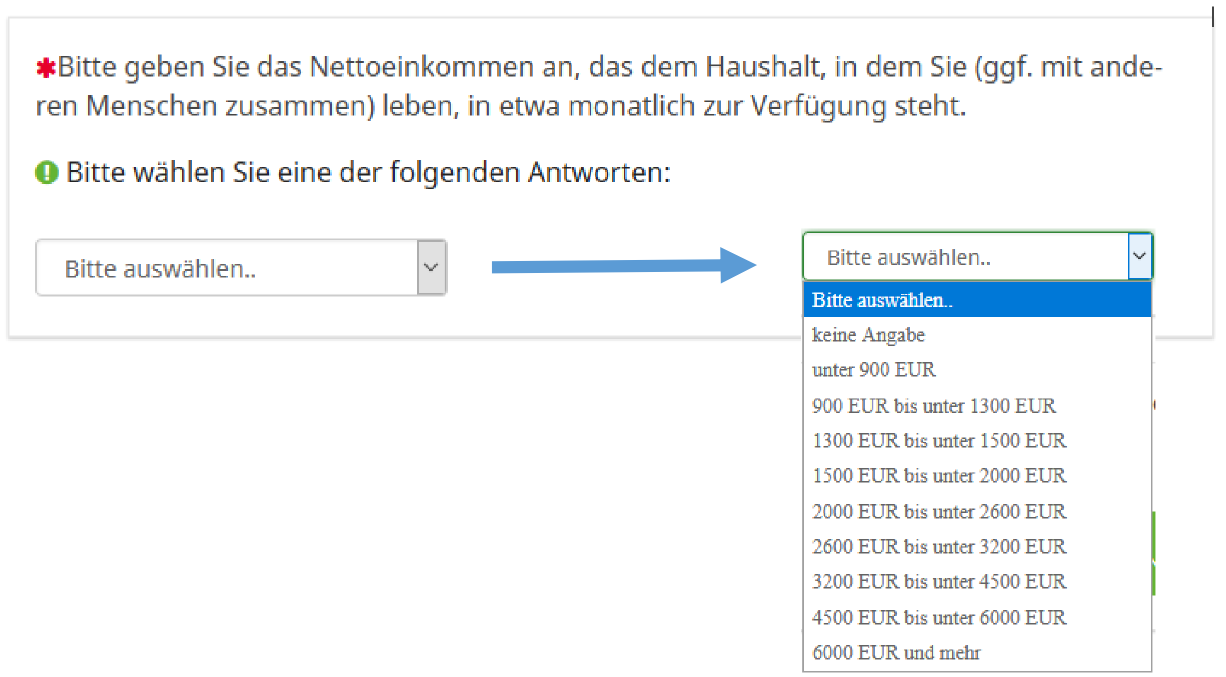

Question/Task	Please indicate the net income that is available to the household in which you live (possibly together with other people) on a monthly basis.
Information	Please choose one of the following answers:
Scale Levels	1; 2; 3; 4; 5; 6 and more.
Item *Income*	
References	German Federal Statistical Office (2018)

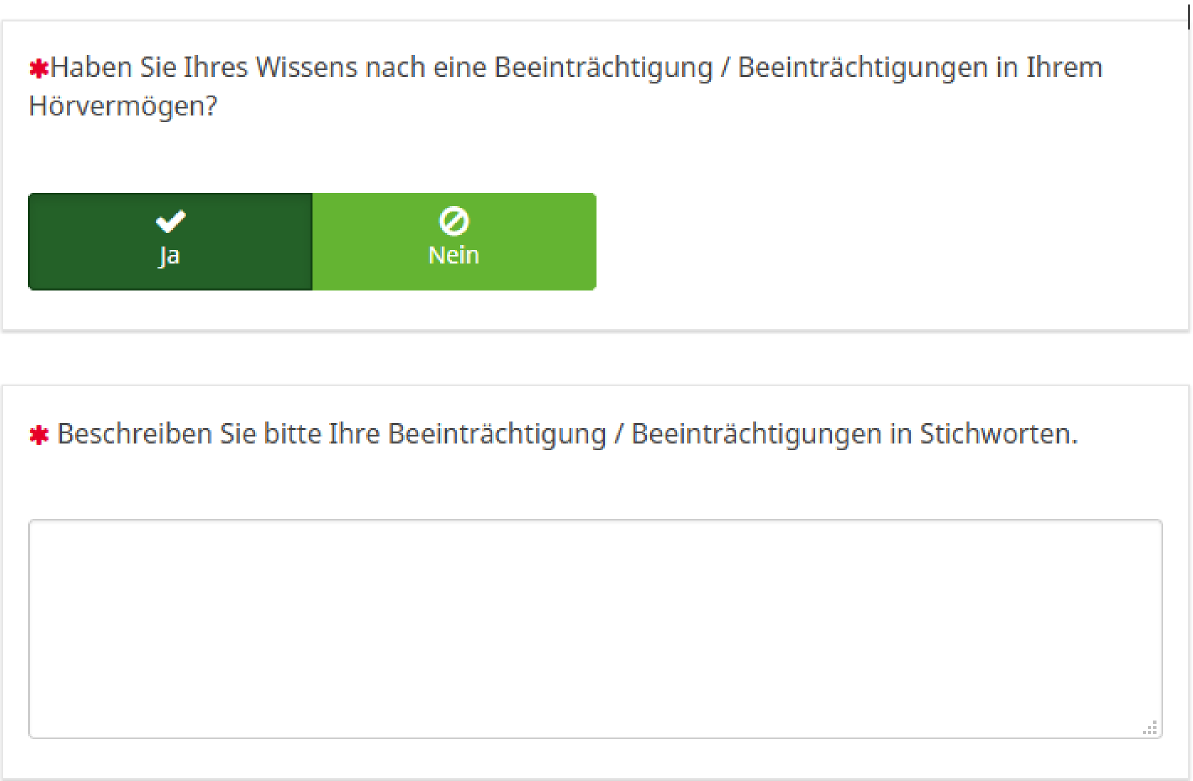
*Haben Sie Ihres Wissens nach eine Beeinträchtigung / Beeinträchtigungen in Ihrem Hörvermögen?

Ja | Nein

* Beschreiben Sie bitte Ihre Beeinträchtigung / Beeinträchtigungen in Stichworten.

Question/Task	To your knowledge, do you have an impairment / impairments in your hearing ability?
Information	[if yes:] Please describe your impairment / impairments in keywords.]
Scale Levels	yes; no; optional free-text description
Item *Hearing Impairment*	

*Wie würden Sie Ihr persönliches Wohnumfeld beschreiben?

	sehr unruhig	unruhig	weder noch	ruhig	sehr ruhig
	○	○	○	○	○

Question/Task	How would you describe your living environment?
Scale Levels	Very lively; lively; neither nor; calm; very calm.
Item *Liveliness*	

*Es sind Ihre ganz persönlichen Meinungen zu den folgenden Aussagen wichtig. Es gibt keine richtigen oder falschen Antworten.

Inwiefern treffen die Aussagen auf Sie zu?

	stimmt genau	stimmt eher	stimmt eher nicht	stimmt gar nicht
1. Sich zu unterhalten macht keinen Spaß, wenn nebenbei das Radio läuft.	○	○	○	○
2. Ich bemerke störende Lärmquellen später als andere.	○	○	○	○
3. Ich vermeide laute Freizeitveranstaltungen wie z.B. Fußballspiele oder Jahrmärkte.	○	○	○	○
4. Ich wache beim geringsten Geräusch auf.	○	○	○	○
5. Ich kann auch in lauter Umgebung schnell und konzentriert arbeiten.	○	○	○	○
6. Wenn ich in der Stadt bin und einkaufe, überhöre ich den Straßenlärm.	○	○	○	○
7. Nach einem Abend in einem lauten Lokal fühle ich mich wie ausgelaugt.	○	○	○	○
8. Wenn ich einschlafen will, stört mich kaum ein Geräusch.	○	○	○	○
9. Am Wochenende bin ich gerne an ruhigen Orten.	○	○	○	○

Question/Task	Your personal opinions on the following statements are important. There are no right or wrong answers.
Information	To what extent do the statements apply to you?
Scale Levels	true; rather true; rather not true; not true.
[To be continued on the next page…]	

[Continuation from the previous page.]	
Item *Noise Sensitivity*	1. Talking is no fun if the radio is on at the same time.<br>2. I notice disturbing noise sources later than others.<br>3. I avoid loud leisure events such as football matches or fairs.<br>4. I wake up at the slightest sound.<br>5. I can work fast and concentrated even in a noisy environment.<br>6. When I am in town and shopping, I overhear the street noise.<br>7. After an evening in a noisy restaurant, I feel exhausted.<br>8. When I want to fall asleep, hardly any sound disturbs me.<br>9. On weekends I like to be in quiet places.
References	Zimmer and Ellermeier (1998)

*Es sind Ihre ganz persönlichen Meinungen zu den folgenden Aussagen wichtig. Es gibt keine richtigen oder falschen Antworten.

Inwieweit treffen die folgenden Aussagen auf Sie zu?

	trifft überhaupt nicht zu	trifft eher nicht zu	weder noch	eher zutreffend	trifft voll und ganz zu
Ich bin eher zurückhaltend, reserviert.	○	○	○	○	○
Ich bin entspannt, lasse mich durch Stress nicht aus der Ruhe bringen.	○	○	○	○	○
Ich gehe aus mir heraus, bin gesellig.	○	○	○	○	○
Ich werde leicht nervös und unsicher.	○	○	○	○	○

Question/Task		Your personal opinions on the following statements are important.<br>There are no right or wrong answers.
Information		To what extent do the statements apply to you?
Scale Levels		does not apply at all; rather not true; neither nor; rather true; completely true
Items	*Extraversion*<br>*Neuroticism*<br>*Extraversion*<br>*Neuroticism*	I am rather reserved.<br>I am relaxed, handle stress well.<br>I am outgoing, sociable.<br>I get nervous easily.
References		Rammstedt and John (2007); Rammstedt et al. (2012)

Assessment of the sounds

In the following, we will deal with the central topic, namely the low-level sounds.

Erinnern Sie sich bitte an leise Geräusche, die Sie in der Vergangenheit in Ihrer Umgebung als leise eingestuft haben.

Nennen Sie zu jedem Geräusch bitte jeweils eine Situation, in der Sie dieses Geräusch gehört haben.

Sie können ein bis drei Geräusche und deren Situationen nennen.
>>> Für die Umfrage ist es besonders wertvoll,
>>> wenn Sie drei persönlich erlebte Geräusche und Situationen angeben würden.
>>>>>>> Es gibt keinen Zeitvorteil bei der Beantwortung des Fragebogens,
>>>>>>> wenn Sie keine eigenen Geräuschsituationen nennen.

Sollten Ihnen mehrere Situationen für ein Geräusch einfallen, nennen Sie das Geräusch mehrfach.
Falls Ihnen keine Geräuschsituation einfällt, lassen Sie bitte alle Felder frei.

	Geräusch	Situation
1	1	a
2	2	b
3		

Question/Task	Please remember sounds that you have classified as low-level in your environment in the past.<br><br>For each sound, please name a situation in which you have heard this sound.<br><br>You can name one to three sounds and their situations.<br>>>> It is especially valuable for the survey,<br>>>> if you would state three personally experienced sounds and situations.<br>>>>>>> There is no time advantage in answering the questionnaire,<br>>>>>>> if you do not name your sound situations.<br><br>If you can think of several situations for one sound, name the sound several times.<br>If you cannot think of any sound situation, please leave all fields blank.
Scale Levels	Free-text descriptions
Items *Sound* and *Situation*	3 sound and 3 situation descriptions.

*Bitte geben Sie an, wie sehr Sie den unten aufgeführten Eigenschaften als Beschreibung des Geräusches "1" zustimmen.

	**stimme sehr zu**	**stimme etwas zu**	**weder, noch**	**lehne etwas ab**	**lehne sehr ab**
angenehm	○	○	○	○	○
chaotisch	○	○	○	○	○
lebendig / pulsierend	○	○	○	○	○
ereignislos	○	○	○	○	○
ruhig	○	○	○	○	○
lästig / störend	○	○	○	○	○
ereignisreich	○	○	○	○	○
monoton	○	○	○	○	○

Question/Task	Please indicate how much you consider the following characteristics as a description of the sound "1".[2]
Scale Levels	strongly agree; rather agree; neither nor; rather disagree; strongly disagree
Items	*Pleasant*<br>*Vibrant/Exciting*<br>*Uneventful*<br>*Calm*<br>*Annoying/Distracting*<br>*Eventful*<br>*Monotonous*
References	ISO (2018)

[2] Instead of "1" the sound description given by the testperson was inserted here and in the following questions.

*Wie bewerten Sie das Geräusch "1" ?

	1	2	3	4	5
tief, dumpf (1) / hoch, schrill (5)	○	○	○	○	○
rauschartig (1) / *tonhaltig (5)	○	○	○	○	○

Tonhaltigkeit eines Geräusches liegt dann vor, wenn innerhalb des Geräusches Einzeltöne zu hören sind

Question/Task	How do you evaluate the sound "1"?
Scale Levels	1; 2; 3; 4;.5
Items	deep, dull (1); high, shrill (5)<br>broadband noise (1); tonal (5)
Information	(The tonal quality of a sound is present when individual tones can be heard within the sound)

*Wie bewerten Sie das Geräusch "1" ?

kaum hörbar		leise		mittel & lauter
○	○	○	○	○

Question/Task	How do you rate the sound "1"?
Scale Levels	scarcely audible (1); [(2)]; low-level (3);<br>[(4)]; middle and louder (5)
Item *Loudness*	

*Was trifft auf die Situation "a" zu?

Die Situation beinhaltet...

	Über-haupt nicht 1	2	3	teils-teils 4	5	6	völlig 7
Arbeit, Aufgaben, Pflichten	○	○	○	○	○	○	○
Intellektuelles, Schöngeistiges, Tiefgründiges	○	○	○	○	○	○	○
Bedrohung, Kritik, Beschuldigung	○	○	○	○	○	○	○
Romantik, Sexualität, Liebe	○	○	○	○	○	○	○
Positives, Erfreuliches, Schönes	○	○	○	○	○	○	○
Negatives, Unerfreuliches, schlechte Gefühle	○	○	○	○	○	○	○
Täuschung, Lüge, Unehrlichkeit	○	○	○	○	○	○	○
Kommunikation, Interaktion, soziale Beziehungen	○	○	○	○	○	○	○

Question/Task	What applies to situation "a"? [3]
Information	The situation contains...
Scale Levels	not at all (1); (2); (3); part-part (4); (5); (6); totally (7)
Items *8 DIAMONDS*: *Duty*<br>*Intellect*<br>*Adversity*<br>*Mating*<br>*pOsitivity*<br>*Negativity*<br>*Deception*<br>*Sociality*	Work, tasks, duties.<br>Intellectual, aesthetic, profound things.<br>Threat, criticism, accusation.<br>Romance, sexuality, love.<br>Positive, pleasant, nice things.<br>Negative things, unpleasant things, bad feelings.<br>Deceit, lie, dishonesty.<br>Communication, interaction, social relationships.
References	S8-II, Rauthmann (2018)

[3] Instead of "a" the situation description given by the testperson was inserted here and in the following questions.

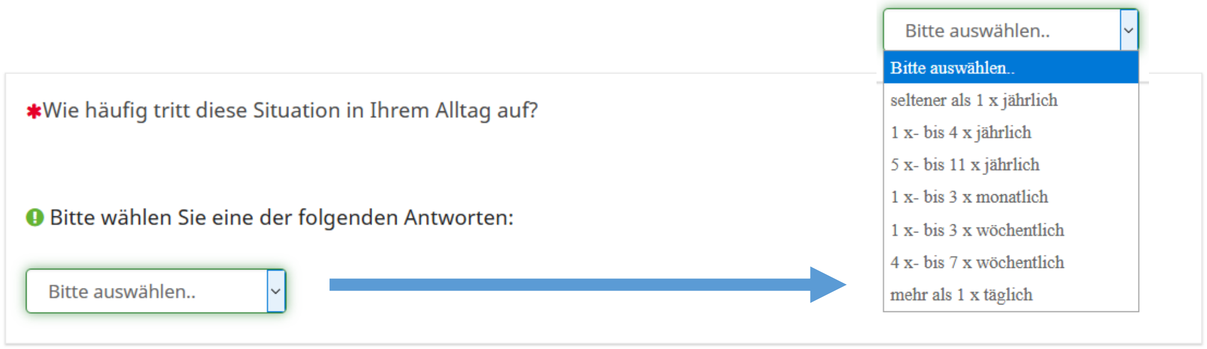

Question/Task	How often does this situation occur in your day-to-day life?
Information	Please choose one of the following answers:
Scale Levels	Less than once a year<br>Once to 4 times per year<br>Five to 11 times per year<br>Once to 3 times monthly<br>Once to 3 times weekly<br>Four to 7 times weekly<br>More than once a day
Item *Frequency*	

*Beurteilen Sie bitte, wie Sie sich in dieser Geräuschsituation fühlen.

[Follow this link to display the 9 pictograms for the evaluation of *Valence*: http://irtel.uni-mannheim.de/pxlab/demos/index_SAM.html]

negativ / positiv ○ ○ ○ ○ ○ ○ ○ ○ ○

*Beurteilen Sie bitte, wie Sie sich in dieser Geräuschsituation fühlen.

[Follow this link to display the 9 pictograms for the evaluation of *Arousal*: http://irtel.uni-mannheim.de/pxlab/demos/index_SAM.html]

ruhig / aufgeregt ○ ○ ○ ○ ○ ○ ○ ○ ○

*Beurteilen Sie bitte, wie Sie sich in dieser Geräuschsituation fühlen.

[Follow this link to display the 9 pictograms for the evaluation of *Control*: http://irtel.uni-mannheim.de/pxlab/demos/index_SAM.html]

schwach / stark ○ ○ ○ ○ ○ ○ ○ ○ ○

Question/Task	*Please assess how you feel in this sound situation* "a"!
Scale Levels	not at all (1); (2); (3); part-part (4); (5); (6); totally (7)
Items *Valence*<br>*Arousal*<br>*Control*	negative / positive<br>calm / excited<br>weak / strong
References	Lang (1980); Bradley and Lang (1994);<br>Pictograms were taken from Irtel (2007)

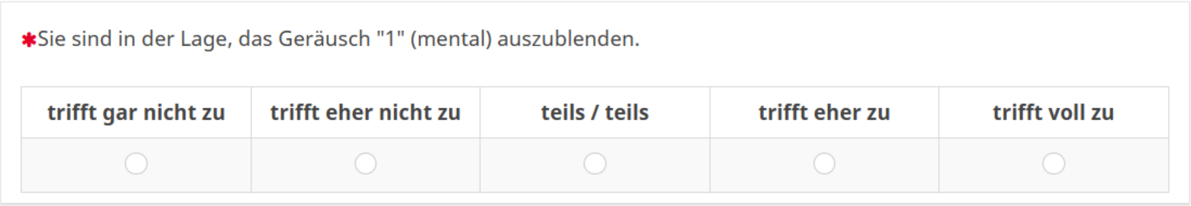
*Sie sind in der Lage, das Geräusch "1" (mental) auszublenden.

trifft gar nicht zu	trifft eher nicht zu	teils / teils	trifft eher zu	trifft voll zu
○	○	○	○	○

Question/Task	Can mentally fade out this sound?
Scale Levels<br>Item: *Specific fade-out*	*does not apply at all*<br>*does rather not apply*<br>*part / part*<br>*does rather apply*<br>does fully *apply* (4))

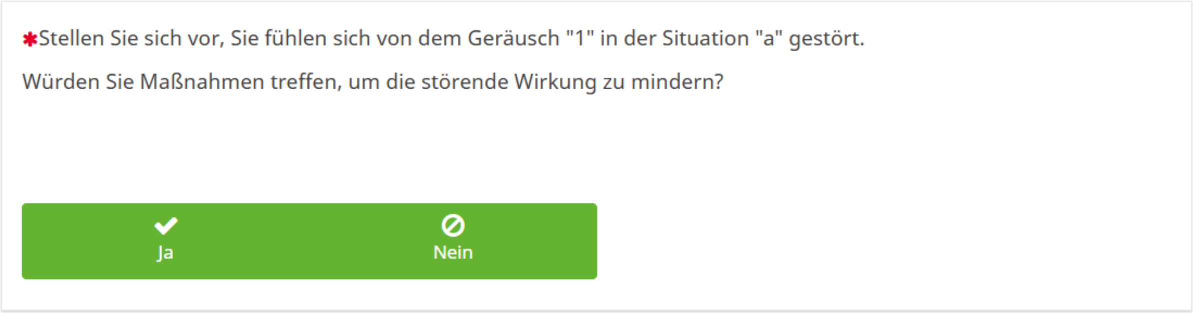
*Stellen Sie sich vor, Sie fühlen sich von dem Geräusch "1" in der Situation "a" gestört.

Würden Sie Maßnahmen treffen, um die störende Wirkung zu mindern?

Ja Nein

Question/Task	Suppose you feel disturbed by the sound 1 in situation a[4].<br>Would you take action to reduce the disturbing effect?"
Scale Levels<br>Item: *Active coping*	*yes; no*

[4] Instead of "1" and "a," the sound and situation descriptions given by the participants were inserted here.

*Sind Sie generell in der Lage, Geräusche (auch laute) mental auszublenden?

trifft gar nicht zu	trifft eher nicht zu	teils / teils	trifft eher zu	trifft voll zu
○	○	○	○	○

Question/Task	*Are you generally able to mentally fade out sounds (even loud ones)?*
Scale Levels<br>Item: *General fade-out*	*does not apply at all*<br>*does rather not apply*<br>*part / part*<br>*does rather apply*<br>does fully *apply* (4))

[The sound-related and the situational questions were repeated for every reported sound situation.]

2. References

Bradley, M. M., and Lang, P. J. (1994). Measuring emotion: The self-assessment manikin and the semantic differential. *Journal of Behavior Therapy and Experimental Psychiatry* 25, 49–59. doi: 10.1016/0005-7916(94)90063-9.

German Federal Statistical Office (2018). "Verteilung der Privathaushalte in Deutschland nach monatlichem Haushaltsnettoeinkommen im Jahr 2017 (in 1.000),". Accessed June 08, 2020. https://de.statista.com/statistik/daten/studie/3048/umfrage/privathaushalte-nach-monatlichem-haushaltsnettoeinkommen/.

Irtel, H. (2007). "PXLab: The Psychological Experiments Laboratory: Version 2.1.11,". Accessed May 06, 2019. http://irtel.uni-mannheim.de/pxlab/demos/index_SAM.html.

ISO (2018). *ISO/TS 12913-2: Acoustics - Soundscape: Part 2: Data Collection and reporting*, no. ISO/TS 12913-2:2018(E). https://www.iso.org/obp/ui/#iso:std:iso:12913:-2:dis:ed-1:v1:en.

Lang, P. J. (1980). "Behavioral treatment and bio-behavioral assessment: Computer applications," in *Technology in mental health care delivery*, ed. J. B. Sidowski, J. H. Johnson, and T. A. Williams (Norwood NJ: Ablex), 119–137.

Rammstedt, B., and John, O. P. (2007). Measuring personality in one minute or less: A 10-item short version of the Big Five Inventory in English and German. *Journal of Research in Personality* 41, 203–212. doi: 10.1016/j.jrp.2006.02.001.

Rammstedt, B., Kemper, C. J., Klein, M. C., Beierlein, C., and Kovaleva, A. (2012). Eine kurze Skala zur Messung der fünf Dimensionen der Persönlichkeit. Big-Five-Inventory-10 (BFI-10). *GESIS-Working Papers* 22.

Rauthmann, J. F. (2018). "S8-II - Eine Ultrakurzversion der Situational Eight DIAMONDS,". Accessed June 08, 2020. https://www.psycharchives.org/bitstream/20.500.12034/620/1/PT_9007482_S8-II_Fragebogen.docx.

Schneider, S. L. (2008). "Applying the ISCED-97 to the German educational qualifications," in *The International Standard Classification of Education (ISCED-97) : An Evaluation of Content and Criterion Validity for 15 European Countries*, ed. S. L. Schneider (Mannheim: MZES), 77–102.

UNESCO Institute for Statistics (2015). *ISCED 2011 operational manual: Guidelines for classifying national education programmes and related qualifications*. Paris: OECD.

Zimmer, K., and Ellermeier, W. (1998). Ein Kurzfragebogen zur Erfassung der Lärmempfindlichkeit. *Umweltpsychologie* 2, 54–63.
